# Spatial single-cell omics: new insights into liver diseases

**DOI:** 10.1136/gutjnl-2024-332105

**Published:** 2025-08-05

**Authors:** Yuan Suo, Robert Thimme, Bertram Bengsch

**Affiliations:** 1 Clinic for Internal Medicine II, University Medical Center Freiburg, Freiburg, Germany; 2 Signalling Research Centres BIOSS and CIBSS, Freiburg, Germany; 3 German Cancer Consortium (DKTK) Heidelberg, Germany, Partner Site Freiburg, Freiburg, Germany

**Keywords:** IMMUNOHISTOPATHOLOGY, PROTEOMICS, METABOLOMICS, LIVER, LIVER IMMUNOLOGY

## Abstract

The liver is a highly multifunctional organ that can perform many metabolic and immunological functions due to a highly complex spatially organised microarchitecture that is disturbed in many liver diseases. Recent methodological advances in spatial omics technologies enable the comprehensive study of the intrahepatic proteome, transcriptome and metabolome at near single-cell and subcellular resolution. The spatial resolution adds an additional dimension to our understanding of the liver, with the potential to revolutionise our insights into the cellular and molecular mechanisms underlying liver physiology and their dysregulation in liver disease. The identification of spatial niches and interactions is empowered by advanced bioinformatics approaches facilitating spatial cellular and network-level analysis and providing novel opportunities for clinical translation. A recent example in immuno-oncology is the use of spatial architecture-based immune classifications, which can improve patient stratification. In this review, we provide an overview of the current methodology and novel spatial insights into metabolic, infectious, immune-mediated, toxic and malign liver diseases and discuss perspectives for clinical translation.

**WHAT IS ALREADY KNOWN ON THIS TOPIC**

- The spatial organisation of the liver microarchitecture is critical for liver function and often altered in liver disease. Histopathologic diagnosis of spatial features is the traditional gold-standard method for clinical diagnosis of many liver diseases.

**WHAT THIS STUDY ADDS**

- This review summarises current advances in spatial profiling technologies with a focus on spatial 'omics' methods and highlights key discoveries that enhance our understanding of liver physiology and pathology.
- It emphasises how spatially defined niches and cell–cell interactions are reshaping current pathophysiologic concepts of liver diseases.

**HOW THIS STUDY MIGHT AFFECT RESEARCH, PRACTICE OR POLICY**

- Spatial omics approaches to liver disease bear high translational potential for improving diagnosis and novel drug target development
- Spatial features of the liver microenvironment can improve patient stratification and clinical decision-making for personalised medicine.

## Introduction

 The liver is centrally involved in metabolism, detoxification and the immune response, playing a crucial role in maintaining systemic homeostasis.^1^ Liver functions are partitioned into different zones of intrahepatic metabolic activity and immune compartmentalisation, thereby highlighting the importance of the spatial organisation of the liver microarchitecture in liver biology and pathology. Within this structured microenvironment, parenchymal hepatocytes, cholangiocytes, endothelial and tissue resident innate immune cells as well as adaptive immune cells form a dynamic network, with interactions determined by spatial organisation and localised signals.^2^ The interplay among these cellular components is fundamental to the liver’s ability to adapt to metabolic demands and respond to external and internal insults. In clinical settings, spatial information is routinely used to understand liver pathology using histochemistry approaches, such as to determine the intrahepatic distribution of immune infiltrates, damage patterns or fibrosis, disrupting physiological liver architecture.^3 4^ Clearly, the understanding of the spatial and functional relationships within the liver microenvironment is essential for uncovering underlying pathophysiological mechanisms, improving diagnostic procedures and patient outcomes.

Recent advancements in single-cell and spatial technologies have revolutionised our ability to investigate tissue biology with remarkable precision. Single-cell sequencing has yielded invaluable insights into cellular heterogeneity and lineage dynamics, facilitating the discovery of novel cell types and states. However, these approaches inherently lack spatial context, a critical aspect for understanding cellular interactions and zonation-specific processes in the liver. In the last decade, omics methods approaching single-cell spatial revolution have evolved. In a seminal study, Halpern *et al* constructed comprehensive genome-wide profiles of hepatocytes by integrating single-cell RNA sequencing (scRNA-seq) with single-molecule RNA fluorescence in situ hybridisation (smRNA-FISH), demonstrating that gene expression patterns correspond with zonal functional differences.^5^ Guilliams *et al* developed a comprehensive spatial proteogenomic atlas encompassing all hepatic cell types in both murine and human tissues by integrating multiple spatial transcriptomic and proteomic approaches.^2^ Notably, spatial transcriptomics was recognised as Nature Method of the Year in 2020, and spatial proteomics received the same honour in 2024, collectively underscoring the transformative impact of spatial technologies on our understanding of tissue organisation and function.^6 7^

This review provides an overview of current spatial profiling technologies covering a broad range of genomic, epigenomic, transcriptomic, proteomic and metabolomic level spatial methods. It discusses key insights from recent studies with translational impact, as well as the challenges associated with these technologies, their applications in liver disease and future research directions to advance our understanding of liver biology in disease.^8^,^13^

## Spatial omics technologies

### Spatial (epi)genomics and transcriptomics

Spatial (epi)genomics and transcriptomics technologies use sequencing-based or imaging-based profiling as their primary detection strategies. Given their shared underlying principles, we address these fields collectively. These methods vary in gene coverage (from a few genes to thousands or the entire genome or transcriptome), whether they employ targeted or untargeted approaches, and spatial resolution (ranging from dozens of cells to cellular and subcellular levels).

#### Imaging-based approaches

**Figure 1:**
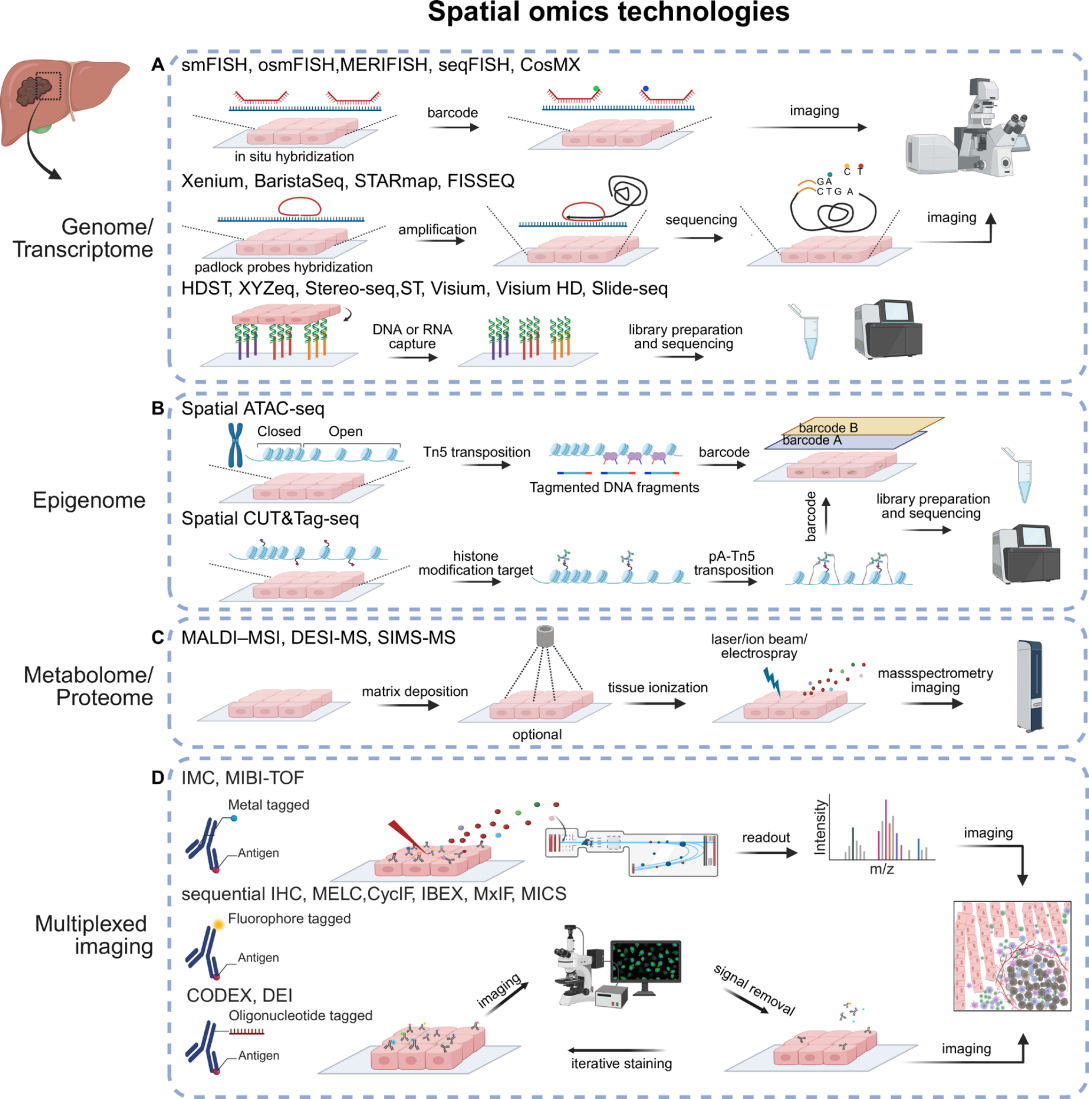
The principles of spatial multiomic technologies. Spatial multiomics approaches are applied to tissue sections for spatial genomics, epigenomics, transcriptomics, metabolomics, proteomics and multiplexed imaging. (**A**) In spatial genomics and transcriptomics, imaging-based technologies use high-resolution microscopes to detect fluorescent signals, either via in situ hybridisation with DNA or RNA targeted by designed probes (eg, smFISH, osmFISH, merifish, seqFISH), or through in situ sequencing which directly sequences nuclei acids within tissue and then tag them with fluorescent signals (eg, Xenium, STARmap and FISSEQ); Sequencing-based technologies capture DNA or RNA from tissue, subsequently sequence and map the data to tissue (eg, HDST, XYZeq, Stereo-seq, Visium, Slide-seq). Spatial epigenomics includes spatial ATAC-seq and spatial CUT&Tag-seq. (**B**) In spatial ATAC-seq, a Tn5 transposition complex accesses open chromatin regions, after which the fragmented DNA is spatially barcoded and sequenced. In spatial CUT&Tag-seq, antibodies targeting histone modification are applied to the tissue, followed by pA-TN5 transposase-mediated tagging of the targeted regions; the resulting DNA is spatially barcoded and sequenced. (**C**) Spatial metabolomics and proteomics include MALDI-MS, SIMS-MS and DESI-MS. These technologies use different ionisation techniques to ionise molecules from tissue sections; the resulting metabolites are analysed by mass spectrometry. Only MALDI-MS requires matrix deposition. Multiplexed imaging is categorised into three groups based on the type of tags attached to antibodies. (**D**) Metal conjugated antibodies, used in CyTOF and MIBI-TOF, enable tissue staining followed by laser or beam-based ablation, where metal isotopes are detected by mass spectrometry. Fluorophore-tagged antibodies, primarily used in immunofluorescence, involve cyclic staining, image acquisition and signal inactivation or removal. Oligonucleotide-tagged antibodies, as in CODEX, allow a one-time antibody binding step followed by iterative cycles of fluorescent labelling, imaging and signal removal. CODEX, codetection by indexing; DEI, DNA exchange imaging; DESI, desorption electrospray ionisation; FISSEQ, fluorescent in situ RNA sequencing; IMC, imaging mass cytometry; MALDI, matrix-assisted laser desorption/ionisation; MIBI, multiplexed ion beam imaging; MICS, MACSima imaging cyclic staining; MS, mass spectrometry; SIMS, secondary ion mass spectrometry; osmFISH, ouroboros single-molecule fluorescence in situ hybridisation; seqFISH, sequential FISH; smFISH, single-molecule FISH.

Imaging-based approaches use high-resolution microscopy to measure the abundance of gene and transcripts by generating fluorescent signals generated after in situ hybridisation (ISH) or in situ sequencing (ISS) approaches (figure 1A).

In ISH, fluorescently labelled probes are used to target DNA or RNA molecules, enabling direct visualisation within the native tissue context. Variations in ISH methods primarily arise from differences in probe design and signal amplification strategies, resulting in diverse approaches to enhance detection sensitivity and specificity. ISH methods exhibit a wide range of gene coverage. For instance, methods like smFISH and ouroboros single-molecule FISH target fewer than 100 genes, while approaches such as multiplexed error-robust FISH, sequential FISH (seqFISH and seqFISH+, ie, Molecular Cartography) and Nanostring CosMX can profile up to 10 000 genes simultaneously.^14^,^18^ ISS uses padlock probes to bind cDNA or mRNA, which are subsequently ligated into circular DNA molecules and amplified via rolling-circle amplification (RCA) to produce micrometre-sized RCA products (RCPs). These RCPs are also used to generate probe-based sequences using methods such as sequencing-by-ligation or sequencing-by-hybridisation. ISS can be further divided into untargeted and targeted methods. Targeted techniques, including 10x Genomics Xenium and STARmap, are inherently limited by the probe numbers and dependence on prior knowledge for panel design. However, newer generations of these methods can now target thousands of genes.^19 20^ Untargeted methods like fluorescent in situ RNA sequencing enable transcriptome-wide analysis, but often suffer from low efficiency.^21^

#### Sequencing-based approaches

Sequencing-based approaches use barcoded oligo (dT) primers to capture poly-adenylated RNA molecules from tissue sections, followed by reverse transcription to generate cDNA. The resulting cDNA is then sequenced to map gene expression profiles back to their spatial coordinates. The resolution of these approaches depends on the size of each capture spot, ranging from 500 µm to subcellular levels (~0.25 µm). In contrast to ISS, sequencing-based technologies perform sequencing on tissues’ mRNA rather than only the sequences embedded in padlock probes. The initial spatial transcriptomics method had a resolution of 100 µm.^22^ The commercialised version, Visium, enhanced to 55 µm, and further improved to a 2 µm spot size with the most recent Visium HD. For capture spots larger than individual cells, computational inference is required to deconvolute mixed cell types. High-resolution methods, such as Seq-Scope (0.5 µm) and Stereo-seq (220 nm), achieve subcellular spatial resolution, allowing mapping of gene expression within individual cells.^23 24^ However, these methods face additional challenges such as data sparsity, cost and difficulties in accurately defining cell boundaries (figure 1A).

Sequencing-based spatial transcriptomics approaches have been successfully integrated with spatial epigenetic assays, such as spatial-ATAC-seq and Spatial-CUT&Tag to simultaneously profile gene expression, chromatin accessibility and epigenetic marks.^25 26^ This integration offers deeper insights into epigenetic regulation within tissue contexts (figure 1B).

### Spatial metabolomics and proteomics

Mass spectrometry (MS) imaging-based technologies are used to determine the spatial distribution and relative abundance of analytes within tissues (figure 1C). These approaches quantify label-free molecules, such as glycans, lipids, proteins and peptides, and are frequently used for metabolomic analysis. Different ionisation techniques define various MS imaging approaches, such as matrix-assisted laser desorption/ionisation (MALDI-MS), secondary ion MS (SIMS-MS) and desorption electrospray ionisation (DESI-MS).^27^,^30^ A common limitation of all these approaches is supracellular resolution and difficulty in identifying specific cell types based on analyte data, emphasising the need for strategies to overlay canonical lineage markers with metabolic tissue maps. Addressing this challenge, a recent study introduces an innovative multimodal approach that integrates MALDI-MS and imaging mass cytometry (IMC), enabling the identification of metabolic heterogeneity at single-cell resolution.^31^

### Multiplexed imaging

Targeted multiplexed imaging can be broadly classified into three groups based on the type of tags attached to antibodies: metal-conjugated, fluorophore-labelled and oligonucleotide-tagged antibody-based methods. These approaches allow antibodies to target proteins, metabolites and RNA, facilitating the integration of multimodal molecular data for a comprehensive understanding of complex biological systems (figure 1D).

#### Metal-based targeted imaging methods

MS-based imaging techniques, such as IMC and multiplexed ion beam imaging (MIBI), use metal-tagged antibodies with different masses to spatially identify 40–50 targets within tissue sections. IMC employs laser ablation to vaporise stained tissue at a resolution of 0.5–1 µm, while MIBI uses ion beam-based secondary ionisation, further improving sensitivity and resolution. However, throughput is limited by acquisition time. While these methods are particularly suitable for tissues with high autofluorescence like the liver that make fluorescence-based readouts more challenging, some signal spillover between metal channels needs to be considered during panel design. While initially developed for the antibody-based detection of proteins and protein modifications, IMC and MIBI have evolved to allow the spatial analysis of RNA, DNA and metabolites, making them invaluable tools for high-resolution multiomic studies^32^,^34^ (figure 1D).

#### Fluorophore-labelled antibody-based methods

Multiplexed immunofluorescence uses fluorophore-tagged antibodies to image multiple targets, enabling the detection of dozens of proteins through a cyclic process involving tissue staining, image acquisition and signal inactivation or removal. Different methods adopt distinct strategies for signal removal between cycles: multiplexed fluorescence microscopy^35^ and iterative bleaching extends multiplexity^36^ rely on chemical removal techniques, multiepitope ligand cartography^37^ uses photo-bleaching, tissue-based cyclic immunofluorescence^38^ combines chemical and physical approaches, and MACSima imaging cyclic staining (MICS)^39^ employs engineered antibodies designed to release fluorescent labels after imaging. These varied approaches enable flexibility and scalability in multiplexed protein detection. However, the iterative processes are time-intensive and may compromise tissue or epitope integrity over multiple cycles (figure 1D).

#### Oligonucleotide-tagged antibody-based methods

Techniques such as DNA exchange imaging^40^ and CODEX (codetection by indexing)^41^ use primary antibodies conjugated with unique DNA oligonucleotides, allowing a one-time antibody binding step followed by shorter iterative cycles of fluorescent labelling, imaging and signal removal. DNA-labelled antibodies can also be combined with signal amplification via sequential hybridisation, as used in Immuno-SABER,^42^ or via tyramide signal amplification^43^ (figure 1D). The key resolution, cost, throughput, strengths and limitations of each technology are summarised in table 1.

**Table 1:** An overview of current spatial technologies

	Tissue type	Resolution	Targets number	Throughput	Cost	Advantage	Limitation
Genome/transcriptome							
Visium	FFPE/FF	55 µm spot, 100 µm c-t-c distance	WT	High	High	User friendly workflow	Lack single cell resolution
Visium HD	FFPE/FF	2×2 µm spot, with no gaps	WT	High	High	Single cell resolution	Expensive, newer technology with limited use cases
Seq-Scope	FF	0.5–0.8 µm	WT	High	Moderate	High resolution	Array preparation may introduce scratches and data loss, especially in liver tissue
Stereo-seq	FF	220 nm spot, 500 or 715 nm c-t-c distance	WT	High	Moderate	High spatial resolution with flexible array size (capture area ranging from 50 mm² to 174.24 cm²)	Limited sensitivity for low-abundance genes
Xenium	FFPE/FF	50 nm	Up to 5000	Moderate	High	Non-destructive; allows post-run IF; high sensitivity	Long imaging time required
HDST	FFPE/FF	2 µm	WT	High	High	Single cell resolution	Data sparsity due to small bead size; reduced sensitivity for low-expressed genes
GeoMx DSP	FFPE/FF	10 µm	WT	High	High	Flexible ROI selection; enables coprofiling of 570+ protein	Measure ROIs, not individual cells
MERFISH	FFPE/FF	Subcellular	Up to 1000	Moderate	High	High resolution	Time-consuming and labour-intensive; requires multiple rounds of hybridisation and imaging
seqFISH+	FF	Subcellular	Up to 10 000	Moderate	high	High resolution	Low detection efficiency; time-consuming due to iterative processes
Nanostring CosMX	FFPE/FF	Subcellular	Up to 6000	Moderate	High	High resolution	Imaging and cyclic hybridisation steps are time-intensive
Epigenome							
Spatial ATAC-seq	FF	10–50 µm	Genome wide	Moderate	High	Chromatin accessibility profiling	Lower efficacy: ~50% information loss
Spatial CUT&Tag-seq	FF	10–50 µm	Genome wide	Moderate	High	Histone modification profiling	Background signal from subnucleosomal DNA
Metabolome/proteome							
MALDI-MSI	FF	10–100 µm	m/z 500–20 000	Moderate	Moderate	High sensitivity; works well with complex tissues	Requires matrix application, which can suppress detection of low-mass analytes; operates under vacuum conditions
DESI-MSI	FF	50–200 µm	m/z 100–2000	Moderate	Moderate	Minimal sample preparation; highly effective for lipidomics	Low resolution
SIMS-MSI	FF	50–100 nm	m/z 1–1000	Moderate	Moderate	High resolution	Limited to low-mass weight compounds
Multiplexed imaging							
IMC	FFPE/FF	1 µm	40	Moderate	Cost~used metal conjugated antibodies	Limited signal overlap	Limited detection of low-expressed proteins
MIBI-TOF	FFPE/FF	0.3–1 µm	40	Moderate	Cost~used metal conjugated antibodies	High resolution	Tissue destruction
mIHC	FFPE/FF	0.2 µm	29	Low	Moderate-low	Cost-effective	Iterative processes
CODEX	FFPE/FF	0.2 µm	60	Moderate	Cost~used oligonucleotide conjugated antibodies	High resolution	Iterative DNA hybridisation; only 3 markers detected per cycle
CycIF	FFPE/FF	0.2 µm	60	Low	Moderate-low	Cost-effective	Requires iterative fluorescence inactivation

CODEX, codetection by indexing; c-t-c, centre-to-centre; CycIF, cyclic immunofluorescence; DESI, desorption electrospray ionisation; FF, fresh frozen; FFPE, formalin-fixed paraffin-embedded; IMC, imaging mass cytometry; MALDI, matrix-assisted laser desorption/ionisation; MERFISH, multiplexed error-robust FISH; MIBI, multiplexed ion beam imaging; MS, mass spectrometry; MSI, MS imaging; m/z, mass-to-charge ratio; ROI, region of interest; SIMS, secondary ion mass spectrometry; WT, whole transcriptome.

## Spatial data analysis

**Figure 2:**
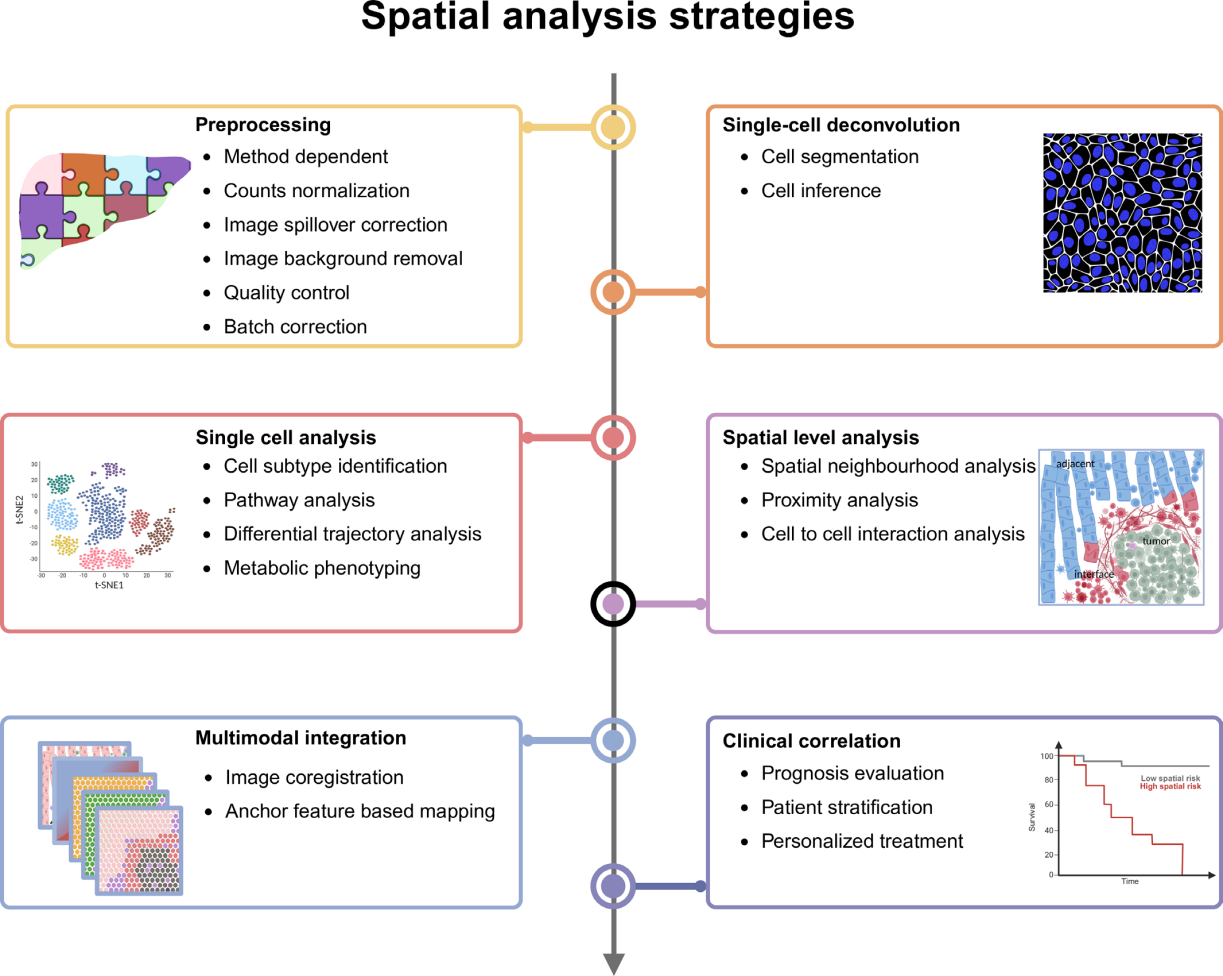
Overview of spatial data analysis. The spatial profiling analysis workflow typically begins with method-dependent preprocessing including batch correction, quality control, normalisation and related steps. Next, the data are segmented into individual cells to enable single-cell analysis. Cell type identification is performed at the cellular level, followed by spatial analysis that characterises cell neighbourhoods and interactions. Multimodal datasets can be integrated via image coordinate registration or anchor feature-based mapping to yield deeper dimensional information. Finally, identified spatial features are correlated with clinical parameters for prognostic or therapeutic insights.

Data analysis for tissue spatial profiling is potentially complex, involving multiple steps tailored to each specific approach. Broadly, the process can be divided into two main steps. The first is methods-dependent data preprocessing, which includes tasks such as image registration and segmentation, sequencing quality control, trimming to generate a gene, protein or metabolite expression matrix aligned to single cells or capture spots. The second is downstream analysis at spatial level, encompassing spatial cell type annotation, spatial expression pattern analysis of genes, proteins or metabolites, multimodal integration, cell-to-cell or gene-to-gene interaction analysis and spatial-temporal trajectory analysis (figure 2). With ongoing advances in bioinformatics, these analyses continue to expand in scope and complexity.

Multiple tools are available for each step of the analysis, and each method may require adaptations depending on the dataset and platform. To streamline these workflows, various pipelines have been developed. For spatial sequencing data, examples include R-based tools such as Seurat,^44^ Giotto,^45^ SPATA^46^ and Selma,^47^ and Python-based tools like Scanpy,^48^ Squidpy,^49^ SPArrOW^50^ and stLearn.^51^ For spatial proteomics, the Steinbock pipeline is commonly used,^52^ while for spatial metabolomics, tools like METASPACE^53^ and others are emerging. These methods are reviewed in more detail elsewhere.^54^,^56^ Frequently used tools and pipelines for liver spatial data analysis are summarised in online supplemental table S1.

## Challenges in data analysis

### Segmentation

For image-based technologies, segmentation is essential to assign transcriptomic or proteomic information to individual cells by defining cell boundaries. A variety of semiautomated^57^,^59^ and fully automated segmentation methods^60 61^ have been developed; however, their accuracy decreases in complex tissues, especially in liver tissue where cells vary substantially in important cell parameters such as size, morphology and the distance between the nucleus and cell boundaries.

### Cell inference

Some spatial transcriptomics technologies lack single-cell resolution, with each spatial spot often encompassing multiple cells. To achieve cell-specific analysis, spot deconvolution methods are employed to identify the constituent cell type within each spot.^62^,^66^ Nevertheless, the accuracy and reliability of such deconvolution analyses depend on the quality of the reference single-cell dataset and do not achieve single-cell level precision.

### Multimodal integration

Given the liver’s critical role in various physiological processes, liver diseases frequently involve complex dysfunctions that require a more integrated analysis. Integrating multimodal datasets for a comprehensive understanding of liver pathologies remains a significant challenge. This difficulty arises from differences in resolution, spatial coverage, throughput and data formats across platforms as well as bioinformatics approaches. Ideally, multiple omics data are generated from the same exact tissue specimen. Recently, a spatial multimodal analysis protocol has been established, combining spatial transcriptomics with MS imaging (MSI) on a single Visium tissue slice, maintaining the sensitivity and specificity of both methods.^67^ Applying multiple analytical technologies to the same tissue section presents technical challenges, as each method often requires specific slide types or processing conditions, which can compromise tissue integrity during subsequent procedures. Alternatively, multimodal integration is often attempted and can be achieved using adjacent sections analysed with different omics technologies;^68^ however, this approach has limitations since the spatial offset may significantly thwart the attempted coregistration. Thus, a major approach is bioinformatic integration on a meta-level that is also suitable for datasets derived from different samples. A common example is spot deconvolution in spatial transcriptomics, where lower-resolution spatial data are integrated with single-cell reference datasets from well-matched samples to infer cell-type composition.

## Spatial insights into liver disease

### Cancer

**Figure 3:**
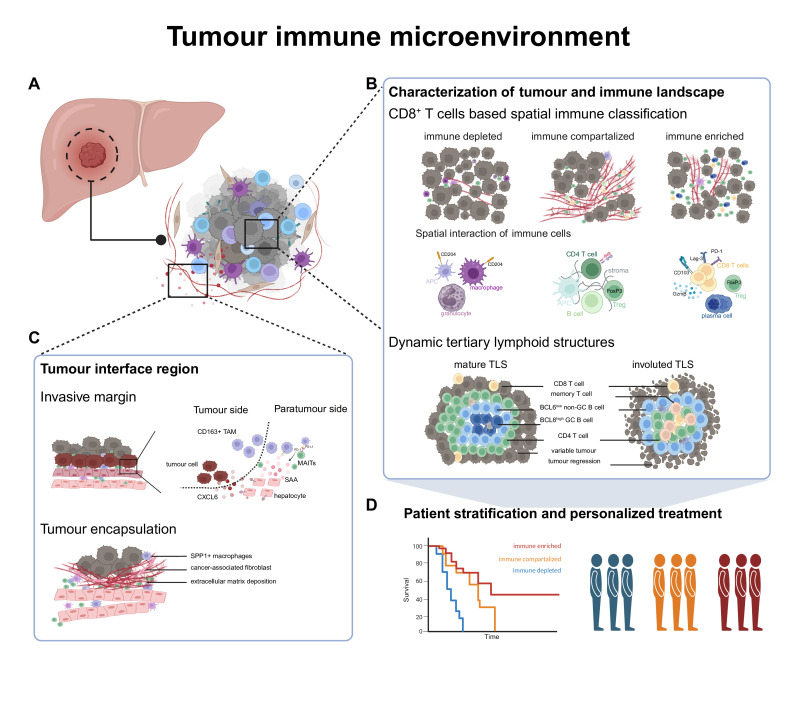
Spatial insights into tumour immune microenvironment. (**A**) Spatial technologies enable the profiling of the tumour immune microenvironment. (**B**) Tumour immune architecture is classified based on CD8^+^T cell infiltration and their interactions with immunoregulatory immune cells, which correlate with ICI treatment response.^84^ Tertiary lymphoid structures exhibit dynamic changes within the microenvironment and are practically identified as CD20^+^CXCL13^+^ lymphoid aggregates in clinical practice.^92^ (**C**) At the tumour interface region, an immune suppressive invasive zone was identified where macrophages are polarised into M2 phenotype, inhibiting MAIT function via PD1/PD-L1.^93 94^ Tumour entity is encapsulated by a fibrotic barrier formed through interactions between macrophages and fibroblasts, which can inhibit immune cell infiltration.^96^ (**D**) The characterised spatial immune architecture enables patient stratification and prognostic prediction. ICI, immune checkpoint inhibitor; MAITs, mucosal-associated invariant T; TLS, tertiary lymphoid structure.

Liver cancer due to primary hepatocellular carcinoma (HCC) or intrahepatic biliary tract cancer/cholangiocarcinoma (iCCA) or metastatic cancers remains a major global health challenge, with HCC ranking as one of the most prevalent causes of cancer-related mortality worldwide^69^ (figure 3A). Molecular analysis has revealed multiple subtypes of these cancer entities. Spatial information is further enhancing our ability to understand the intra and interpatient heterogeneity of liver cancers. Immunotherapy with immune checkpoint inhibitors (ICI) has become the main systemic treatment option for primary liver cancer.^70^,^75^ Patient responses to these therapies vary largely, with objective responses between 20% and 35% and some patients achieving complete remission. While previous single-cell methods using single-cell RNAseq or cytomics already indicated a role for the immunological landscape in patient outcomes to HCC,^76^,^83^ highlighting important roles for distinct innate and adaptive immune populations, context was missing. Spatial methods now enable a more precise understanding of the relationship between cellular interactions, the immune architecture and outcome in these liver cancers. As discussed below, spatial immune architecture-based tumour classifications may allow the prediction of patient outcome to immunotherapy with implications for patient stratification and personalised treatment decisions.

## Tumour immune microenvironment

The tumour microenvironment harbours variable amounts of different immune cell populations of the innate and adaptive immune system such as granulocytes, monocytes, macrophages, natural killer cells (NKs), dendritic cells (DCs), B cells and T cells. Spatial profiling methods underscore the relevance of the tumour immune infiltrate for patient prognosis. Comprehensive spatial profiling of immune cell subsets in HCC tumours revealed significant heterogeneity of immune subset infiltration across patients, with CD8^+^ T cells as an important surrogate for immune infiltration^84^ (figure 3B). Tumours with significant immune infiltration further varied by degree of interacting immunoregulatory immune cell populations and compartmentalisation of immune cells to the tumour stroma, informing about three major immune architectures that can be classified as—immune depleted (low immune infiltration), compartmentalised (preferential retention of immune cells in the tumour stroma with a prominent role for myeloid cell-T cell interactions) and enriched (highest accumulation of CD8^+^ T cells in tumour parenchyma). This spatial immune architecture-based classification of HCCs was validated in a cohort of patients treated with ICIs, highlighting the longest survival for patients with an enriched spatial immune class.^84^ In the enriched immunotype, there is a pronounced accumulation of CD8^+^ T cells that express high levels of immune checkpoints, suggesting a correlation of the enhanced responsiveness to PD-1-targeted ICI therapies.^84^ Different immunotherapy strategies may be required for patients with other immunotypes. In non-responders to combined ICI using anti-CTLA4 and anti-PD-L1, the tumour centre displayed enhanced interactions of Tregs and CD8^+^ T cells, suggesting that the spatial organisation of these immune cells can serve as a negative prognostic marker.^85^ Recently, a NK cell-associated spatial immune score system was developed to predict recurrence risk in HCC, based on the spatial expression pattern of SPON2, ZFP36L2, ZFP36, VIM and HLA-DRB1.^86^ NK cell enrichment at the invasive zone was found to be correlated with lower recurrence risk, possibly due to their interaction with CD8^+^ T cells which contributes to antitumour immunity.^86^ Further prospective studies are required to evaluate the utility of spatial classification approach as a predictive biomarker for therapy. Of note, these data further refine the concept of ‘hot’ (ie, immune-infiltrated) tumours as suitable targets for checkpoint therapies which is shared across many cancer entities. Spatial single-cell interactions can help further resolve the immune mechanisms in enriched tumours. Magen *et al* demonstrated the presence of intratumoural pathogenic triads consisting of DC–CD4^+^ T helper cell–CD8^+^ T cell niches that were associated with checkpoint therapy response within enriched tumours, highlighting further spatial immune correlatives of response.^87^ Moreover, several studies inform about the relevance of immune aggregates and tertiary lymphoid structures (TLSs) that are identifiable by spatial analysis. Multiplexed imaging and spatial transcriptomics performed in HCC patients undergoing combination treatment with cabozantinib and nivolumab revealed that clinical responses were associated with increased immune cell infiltration, and particularly an enrichment of various lymphoid aggregate structures within the TME, features that are not identifiable by suspension-based single-cell methods.^88 89^

TLSs—defined based on aggregates of T cells and B cells—have emerged as critical predictors of immunotherapy efficacy across multiple cancers, as revealed by multiomics spatial profiling studies.^90 91^ In HCC, Shu *et al* recently demonstrated that TLSs can serve as both prognostic biomarkers and predictors of immunotherapy response.^92^ Interestingly, multiplexed spatial profiling combined with examination of T cell and B cell repertoires reveals that TLSs are dynamic structures that display distinct morphologies and functional associations with T cells. Given the challenges of routinely detecting TLSs in fine needle aspirates, Shu *et al* further identified CD20^+^CXCL13^+^ lymphoid aggregates as surrogate markers for TLSs, thereby offering a practical approach to predict immunotherapy outcomes^92^ (figure 3B). The precise function and organisation of TLS remain to be fully characterised. Future studies incorporating spatial immune structures into prospective clinical trials will be essential for refining predictive biomarkers and identifying novel immunotherapeutic targets in HCC.

## Tumour interface region

The tumour margin is considered a critical interface for tumour–immune crosstalk, serving not only as a physical boundary but also as a dynamic hub of cellular interactions that regulate immune evasion, stromal remodelling and tumour progression. Spatial technologies have been instrumental in decoding the cancer ecosystem at this important region. For example, Wu *et al* using Stereo-seq defined a 500 μm wide invasive zone in HCC, revealing mechanisms by which tumour cells sustain local immunosuppression.^93^ They showed that tumour cell-derived CXCL6 induces hepatocyte overexpression of serum amyloid A proteins, which in turn recruit and polarise M2 macrophages, amplifying immune suppression^93^ (figure 3C). Hepatocytes are frequently underrepresented in scRNA-seq studies; in contrast, spatial technologies preserve tissue context, thereby enabling comprehensive analysis of these key parenchymal cells. Spatial proteomic and imaging technologies have elucidated functional cellular interactions within the margin area. Tumour-associated macrophages (TAMs) were found to engage with mucosal-associated invariant T (MAIT) cells, impairing their cytotoxic function and blocking their intratumoural migration.^94^ Disrupting MAIT-TAM interactions restores antitumour activity, highlighting this axis as a potential therapeutic target^94^ (figure 3C). These findings illustrate how the invasive margin orchestrates immunosuppressive networks, but another key histopathological feature further adds to the complexity: tumour encapsulation, which is observed in up to three out of four HCC patients.^95^ Recent spatial transcriptomic analysis in anti-PD-1-treated HCC patients identified a fibrotic tumour immune barrier enriched with SPP1^+^ macrophages and cancer-associated fibroblasts (CAFs).^96^ SPP1^+^ macrophages activate CAFs, driving extracellular matrix (ECM) deposition that physically restricts T cell infiltration and diminishes immunotherapy efficacy^96^ (figure 3C). Preclinical models further confirm that inhibiting SPP1 or specifically deleting Spp1 in macrophages increases the density of tumour-infiltrating lymphocytes and enhances the effectiveness of anti-PD-1 therapy.^96^ These findings underscore the potential of spatially defined targets in the margin region to restore immune activity and improve therapeutic outcomes. Nevertheless, the heterogeneity of the microenvironment in the HCC margin region requires further investigation.

## Cirrhosis and fibrosis

Hepatic fibrosis and cirrhosis are the common end-stage outcomes of chronic liver inflammation driven by viral infections, toxic insults, metabolic dysfunction or autoimmune processes. Persistent inflammatory stimuli active across multiple signalling pathways lead to the excessive deposition of ECM, finally resulting in architecture disruption, organ dysfunction and eventual organ failure.^97^ Clinically, eliminating the underlying inflammation is the major strategy while the development of novel antifibrotic therapies remains a challenge. Spatial technologies allow for the dissection of the heterogeneous cellular and molecular microenvironment niches that drive fibrogenesis.

## Hepatic stellate cell in fibrogenesis

Hepatic stellate cells (HSCs) have been traditionally perceived as primary drivers of hepatic fibrosis but are now increasingly recognised for their roles as active regulators of liver homeostasis, regeneration and cancer progression. Recent work demonstrates that HSCs maintain hepatocyte zonation and metabolic function through RSPO3-mediated WNT signalling modulation, and their loss exacerbates steatotic liver disease. They also exhibit distinct influences on tumour development, with cytokine-producing HSCs protecting against hepatocyte death and HCC development, whereas myofibroblastic HSCs enhance tumour growth by promoting collagen deposition and matrix stiffness.^98^,^100^ The activation of HSCs and subsequent differentiation into myofibroblasts that drive the pathological deposition of ECM is a key process in liver fibrosis.^101^ HSC activation is induced by a complex interplay of intracellular and extracellular signals.^102^ Once activated, HSCs maintain their fibrogenic function through autocrine signalling, a process supported by increased direct cell-cell contacts, which represent potential therapeutic targets.^103^ However, clinical trials targeting HSC activation pathways have been hampered by systemic toxicity, underscoring the need for spatially precise strategies that target fibrogenic niches rather than the entire liver.^104 105^ Since fibrosis is a locally regulated process, spatial high-resolution methods have advantages to study the relevant microenvironments.

**Figure 4:**
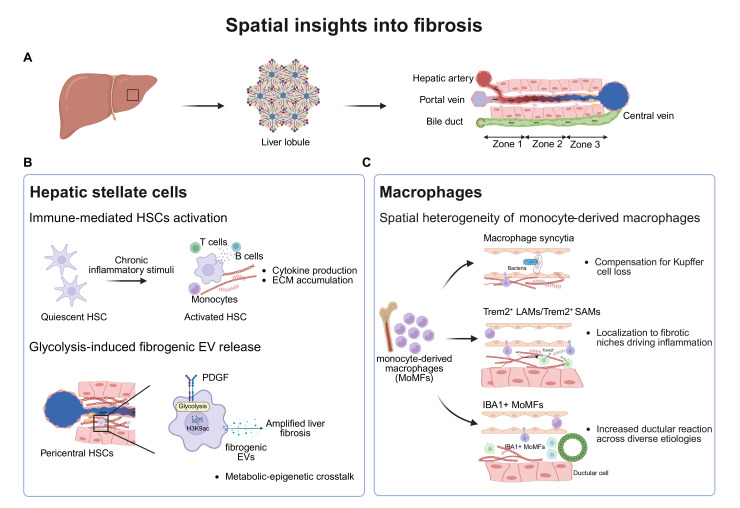
Spatial insights into liver fibrosis. (**A**) The liver is organised into hexagonal lobules. Within each lobule, periportal hepatocytes (zone 1) are at the portal triad region, followed by mid-lobular cells (zone 2), and pericentral hepatocytes surround the central vein (zone 3). (**B**) In fibrosis and cirrhosis, spatial profiling reveals differential gene expression, with immune cells enriched in fibrotic region which induce hepatic stellate cell activation.^106 107^ Pericentral HSCs exhibit increased glycolysis, promoting fibrogenic EV release via epigenetic mechanisms.^108^ (**C**) As fibrosis progresses, bone marrow-derived macrophages form compensatory syncytia to capture bacteria, compensating for the loss of KCs.^113^ Bone marrow-derived monocytes exhibit heterogeneity under the influence of localised signal.^118 119^ EV, extracellular vesicle; HSCs, hepatic stellate cells; KCs, Kupffer cell; LAMs, lipid-associated macrophages; MoMFs, monocyte-derived macrophages; SAMs, scar-associated macrophages.

Integrating spatial transcriptomics with scRNA-seq, Li *et al* and Chung *et al* localised and characterised cell types and gene expression patterns in fibrotic regions.^106 107^ These studies demonstrated that fibrogenesis is not uniform but is spatially compartmentalised, with parenchyma and fibrosis regions exhibiting distinct transcriptional programmes and cellular compositions.^107^ Moreover, fibrotic regions exhibit localised immune cell enrichment, particularly monocytes, B cells and T cells, suggesting that immune-mediated HSC activation occurs in spatially defined microenvironments. Further detailed spatial analyses are required to precisely determine the pathogenic or protective roles of these immune cell populations^106^ (figure 4B).

Beyond cellular interactions, spatial technologies have also enabled the discovery of metabolic heterogeneity within HSC populations. Activated HSCs exhibit zone-specific metabolic shifts, with pericentral HSCs displaying increased glycolysis, which promotes the release of fibrogenic extracellular vesicles (EVs) and upregulates EV-related gene expression through epigenetic mechanisms^108^ (figure 4B). These metabolic alterations were uncovered using spatial transcriptomics, which mapped glycolysis-related gene expression to specific fibrotic regions. Such insights emphasise the necessity of targeted antifibrotic therapies rather than systemic interventions.

## Macrophages in fibrogenesis

Hepatic macrophages, comprising tissue-resident Kupffer cells (KCs) and monocyte-derived macrophages (MoMFs), exhibit spatial and functional heterogeneity, adapting dynamically to distinct liver microenvironments.^2 109^ These spatially restricted macrophage populations play critical roles in regulating inflammation, responding to tissue injury and driving fibrogenesis, but also contribute to fibrosis resolution.^110^,^112^ During liver fibrosis, KCs lose their identity and function, with MoMFs compensating for this loss. Using high-resolution intravital microscopy (IVM), Peiseler *et al* demonstrated that fibrogenesis involves progressive liver vasculature remodelling and KC maladaptation, which is compensated by bone marrow-derived monocytes forming KC-like syncytia to capture bacteria.^113^ MoMFs differentiate into specialised subsets, such as ‘lipid-associated macrophages’ (LAMs) or ‘scar-associated macrophages’, which are defined by the expression of TREM2, CD9 and osteopontin, and localise within fibrotic niches.^114^,^117^ Spatial transcriptomics further identified TREM2^+^ macrophages accumulating specifically at sites of collagen deposition, inflammation and ECM remodelling, confirming that these cells form structured fibrotic niches. Additionally, systemic soluble TREM2 levels correlate with metabolic dysfunction-associated steatohepatitis (MASH) severity, suggesting that spatially mapped TREM2^+^ macrophages serve as important regulators of both metabolic dysfunction and fibrosis progression.^118^ Across different aetiologies, spatial technologies have revealed that fibrosis progression is characterised by the organised accumulation of IBA1^+^ MoMFs near CK19^+^ ductular cells, a pattern that single-cell approaches alone would not be able to detect.^119^ This spatial proximity suggests crosstalk between MoMFs and ductular cells, reinforcing a key immune-fibrotic axis. These findings revealed the necessity of spatial technologies in uncovering dynamic immune-stromal interactions in fibrosis, providing new opportunities for antifibrotic therapies (figure 4C).

## Immunity and infection

Positioned at the crossroads of the gut and systemic circulation, the liver is continuously exposed to microorganisms and endotoxins under normal physiological conditions. To prevent excessive inflammation while maintaining immunosurveillance, the liver adopts a unique tolerogenic microenvironment. This balance is essential for sustaining liver function and maintaining systemic homeostasis.^120^ Most studies on infection-induced liver pathology have predominantly relied on peripheral blood analyses, which provide only limited insight into intrahepatic immune responses and fail to capture the spatial organisation of host–pathogen interactions within liver tissue. This highlights the need for spatial multiomic analyses, to precisely map immune cell localisation, pathogen distribution and cellular interactions within native hepatic microenvironment.

## Hepatitis B virus infection

**Figure 5:**
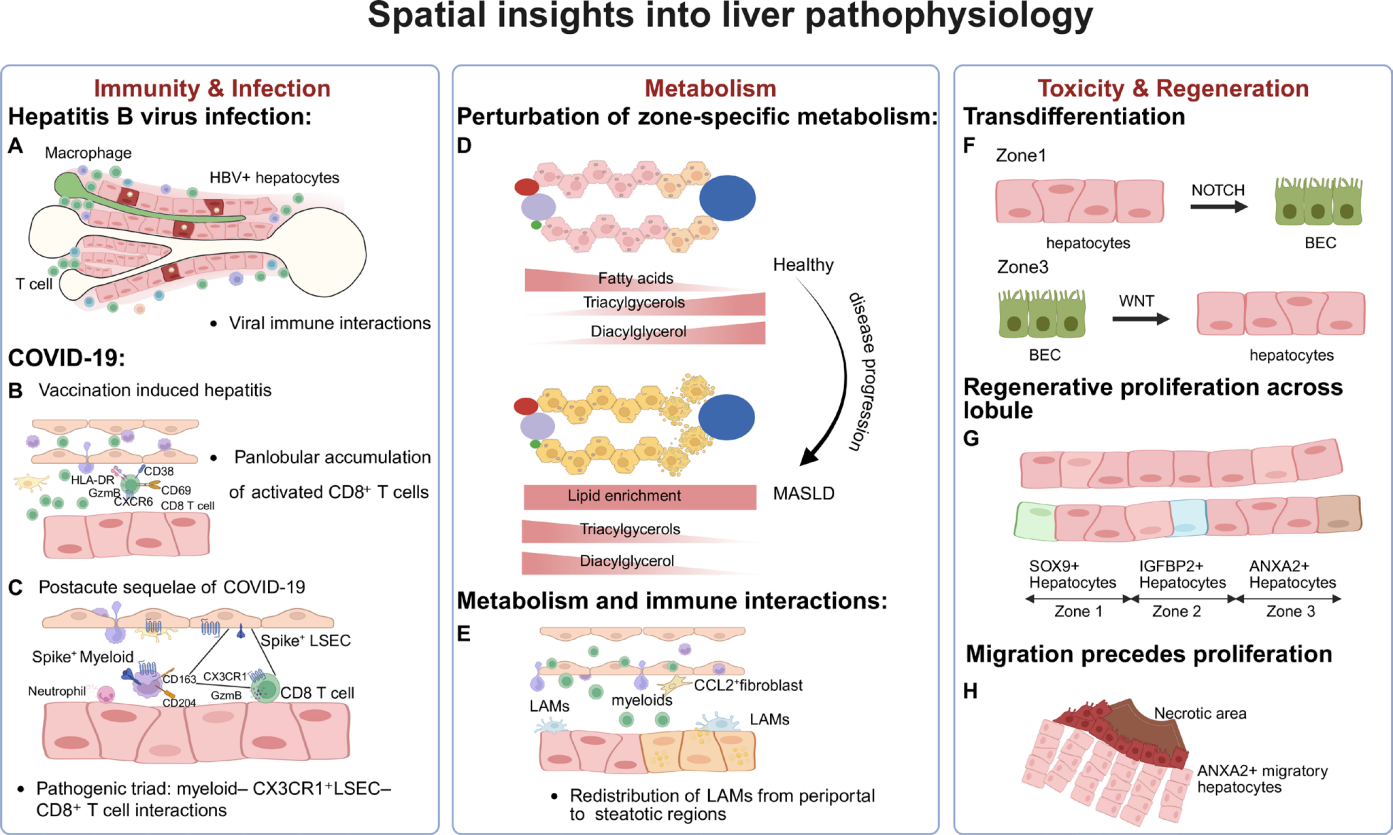
Spatial insights into infection, metabolic diseases and liver regeneration. (**A**) In HBV infection, virus–immune interactions correlate with liver injury.^122^ (**B**) In COVID-19, vaccination-induced hepatitis is primarily enriched with CD8^+^ T cells.^131^ (**C**) In postacute sequelae of COVID-19, a pathogenic cellular triad is identified, comprising interactions among CD8^+^ T cells, M2 myeloids and CX3CR1^+^ LSECs.^133^ (**D**) In metabolism-related disease, techniques such as MALDI-MS and DESI-MS identify the redistribution of metabolites during disease progression.^141 142^ (**E**) In steatosis, LAMs are redistributed to the steatotic region, where they interact with the fibroblast and promote the disease progress.^2^ (**F**) During liver toxicity and regeneration, hepatocytes and BECs in different zones exhibit different transdifferentiation mechanisms.^146 147^ (**G**) Regenerative proliferating hepatocytes show regional heterogeneity, with SOX9^+^ and IGFBP2^+^ proliferating cells predominantly found in zones 1 and 2, respectively, (**H**) ANX2^+^ cells in zone three migrate to the injured interface prior to proliferation.^148^,^150^ BECs, bile epithelial cells; DESI, desorption electrospray ionisation; HBV, hepatitis B virus; LAMs, lipid-associated macrophages; LSECs, liver sinusoidal endothelial cells; MALDI, matrix-assisted laser desorption/ionisation; MS, mass spectrometry.

Hepatitis B virus (HBV) infection remains the most prevalent, affecting over 250 million individuals globally. As a non-cytopathic virus, HBV-induced liver injury is primarily immune-mediated, yet the spatial organisation of host–virus interactions within the hepatic microenvironment remains poorly characterised.^121^ Emerging spatial multiomic, approaches are now dissecting this complexity. IMC analysis with 30-plex panels has mapped the immune landscape in chronic HBV, revealing that immune-active patients exhibit significant enrichment of activated CD8^+^ T cells and macrophages in lobular regions, correlating with elevated alanine-aminotransferase (ALT) levels^122^ (figure 5A). However, the precise spatial interactions between infiltrating immune cells and HBV-infected hepatocytes remain unresolved.

Multiplex immunofluorescence has uncovered HBV antigen zonation patterns: HBV core^+^ hepatocytes frequently appear as isolated cells, while HBsAg^+^ hepatocytes cluster into dense aggregates.^123 124^ Visium spatial transcriptomics further refined these findings, revealing that HBV genome integration contributes to HBsAg expression in clustered hepatocytes, while covalently closed circular DNA (cccDNA) remains the dominant source of viral protein production, particularly core protein.^125^ However, the 55 µm spot resolution of Visium technology captures multiple cells per spot, preventing single-cell resolution and precise assignment of viral integration or immune interactions to specific cell types. Future integration of single-cell spatial multiomics will be essential to precisely define immune-viral microenvironment niches and uncover mechanisms of immune evasion and immunopathology.

## COVID-19

COVID-19 infection has been associated with hepatic injury, with spatial and single-cell transcriptomics offering critical insights into disease-specific liver pathology.^126 127^ While vaccination remains key to control the COVID-19 pandemic, cases of postvaccination liver inflammation have been reported.^128^,^130^ IMC was applied to investigate immune-mediated liver injury following COVID-19 vaccination, identifying a CD8^+^ T cell-dominated infiltration, which was distinct from the CD4^+^ T cell and B cell aggregates characteristic of autoimmune hepatitis (AIH).^131^ (Figure 5B) Uzun *et al* further validated these findings through the integration of spatial transcriptomics and CODEX multiplexed imaging, revealing upregulated oxidative phosphorylation in hepatocytes in COVID-19 vaccine-induced liver injury, connecting CD8^+^ T cell infiltration to hepatocyte metabolic stress, while AIH exhibited increased interferon-driven inflammatory response.^132^

In a recent study, acute hepatitis of unknown origin in children following COVID-19 infection was investigated.^133^ Using IMC, a distinct immune infiltration pattern and persistence of SARS-CoV-2 viral proteins were identified in the liver, indicating the hepatitis as a postacute sequel of COVID-19 (PASC). Spatial profiling identified a pathogenic triad comprising CX3CR1^+^ CD8^+^ T cells, M2-like myeloid cells, neutrophils and CX3CR1^+^ liver sinusoidal endothelial cells (LSECs) localised within injured regions. These cells formed specific cellular neighbourhoods associated with severity of hepatitis (figure 5C). These findings highlight the distinct immune mechanisms underlying liver inflammation in different contexts and emphasise the need for advanced tools to dissect and monitor infection-related liver damage.

## Metabolism

The liver’s metabolic functions are spatially compartmentalised across its lobules, with distinct zones specialising in specific metabolic processes. The periportal (zone 1) region prioritises oxidative metabolism, including gluconeogenesis and β-oxidation, while the centrilobular (zone 3) region specialises in lipogenesis, detoxification and glycolysis. Halpern *et al* combined smFISH and scRNA-seq to map zonate gene expression, revealing that ~50% of hepatic genes exhibit spatial compartmentalisation correlated to metabolic roles.^5^ However, these studies focused on transcriptional zonation, leaving organelle-level heterogeneity underexplored. Kang *et al* advanced this field integrating spatial proteomics, IVM and functional assays, demonstrating that mitochondrial heterogeneity—varying in size, membrane potential and enzymatic activity—is aligned with metabolic zonation.^134^ This spatial organisation ensures metabolic efficiency but becomes disrupted in diseases like metabolic dysfunction-associated steatotic liver disease (MASLD) and MASH. While scRNA-seq and snRNA-seq have identified dysregulated pathways including lipid metabolism and immune activation, they lack spatial context to resolve zonate injury patterns.^135^,^138^

## Perturbation of zonate metabolism

Spatial metabolomics technologies, such as MALDI-IMS and DESI-MSI, have emerged as important tools for resolving localised metabolic perturbations in disease pathogenesis. MALDI-IMS has revealed correlations between fucosylated N-glycan structures and fibrosis severity during NAFLD/NASH development, highlighting N-glycan alterations in fibrotic liver tissues.^139^ MALDI-IMS typically requires matrix application, which introduces analytical constraints for small metabolites; however, DESI-MSI operates under ambient conditions, offering unique advantages for lipidomic profiling. DESI-MSI has mapped lipid zonation within hepatic lobules, showing fatty acids predominate periportally and triacylglycerols (TAGs) pericentrally, reflecting their metabolic roles.^140^ During MASLD progression, this zonation becomes disrupted, with lipid accumulation, including diacylglycerol (DAG) and TAG, shifting from pericentral to periportal zones as the disease advances to MASH^141 142^ (figure 5D). The perturbation of liver zonation has been further validated through integration of spatial transcriptomics, bulk RNA sequencing and ISH, implicating the Wnt signalling pathway as a key regulator of liver metabolism.^143^ While MALDI-IMS and DESI-MSI provide complementary insights into metabolic dynamics, their integration with emerging spatial single-cell methodologies will be critical for dissecting cell type specific metabolic dysregulation.

## Metabolism and immune interactions

During the progression of MASLD/MASH, hepatic macrophages undergo spatial and functional changes, dynamically adapting their localisation and phenotypic states in response to evolving metabolic and inflammatory cues. Guilliams *et al* employed spatial proteogenomics to construct a spatial atlas of all hepatic cells, identifying a disease-driven redistribution of LAMs from bile ducts to pericentral steatotic regions as MASLD progresses. This shift was revealed to be driven not by direct cell–cell interactions but by local metabolite exposure. By preserving tissue architecture, spatial technologies allowed researchers to pinpoint the exact hepatic microenvironments where LAMs undergo metabolic reprogramming, reinforcing their role in disease progression^2^ (Figure 5E). Similarly, Guillot *et al* used spatial transcriptomics and imaging to map the accumulation of IBA1^+^ MoMFs in periportal regions and areas of ductular reaction, demonstrating that their presence strongly correlates with disease severity across MASLD and MASH.^119^ These macrophages exhibited distinct phenotypic markers, particularly when adjacent to CK19^+^ ductular cells, suggesting functional interactions that contribute to disease progression. While further functional analysis is required in their native tissue environment, spatial technologies are essential for dissecting immune-metabolic crosstalk and its contribution to disease pathogenesis.

## Toxicity and regeneration

The liver employs unique regenerative mechanisms to maintain the liver-to-bodyweight ratio essential for homeostasis.^144^ This regenerative capacity is primarily driven by hepatocyte proliferation and transdifferentiation between bile duct epithelial cells (BEC) and hepatocytes.^145^ However, regeneration is not uniform across the liver, and recent single-cell analyses have uncovered functional heterogeneity among proliferative hepatocytes. Multimodal spatial approaches now provide critical spatial and temporal insights into the complex dynamics of liver regeneration.

## Transdifferentiation

The process of transdifferentiation in the liver follows distinct zonal patterns that are spatially regulated, underscoring the necessity of spatial technologies to dissect these regenerative mechanisms. Transdifferentiation into BECs occurs predominantly in periportal hepatocytes and is driven by NOTCH signalling.^146^ However, zone 3 hepatocytes rarely undergo transdifferentiation into BECs, even in the presence of NOTCH activation, suggesting a spatially restricted regulatory barrier that prevents hepatocyte plasticity in this region.^146^ When hepatocyte-mediated regeneration is impaired, BEC-to-hepatocyte transdifferentiation occurs, which is regulated by WNT/β-catenin signalling^147^ (figure 5F). Traditional bulk and single-cell sequencing approaches fail to capture these region-specific transdifferentiation dynamics, as they lack spatial resolution. Spatial transcriptomics and multiplex imaging technologies have been instrumental in mapping the precise localisation of transdifferentiating cells, revealing how zonal signalling dictates cell fate decisions in liver regeneration.

## Regenerative proliferation

Hepatocyte proliferation within the liver lobule is highly heterogeneous, regulated by distinct subpopulations that are spatially confined. In zone 1, SOX9^+^ hepatocytes expand throughout the lobule to facilitate liver repair following injury, without giving rise to tumours.^148^ In zone 2, IGFBP2^+^ hepatocytes exhibit enhanced proliferative capacity, acting as a source for replenishing both periportal (zone 1) and pericentral (zone 3) hepatocytes after regenerative stimuli such as partial hepatectomy or pregnancy^149^ (figure 5G). Recent research has uncovered a previously unrecognised regenerative mechanism, in which hepatocyte migration precedes proliferation. This discovery, enabled by the integration of snRNA-seq, spatial transcriptomics, IVM, and smFISH, identified a distinct ANXA2^+^ subpopulation of migratory hepatocytes that actively relocate to injury sites before initiating proliferation.^150^ Thus, the application of spatial transcriptomics and live imaging has fundamentally enhanced our understanding of the cellular mechanisms of liver regeneration (figure 5H).

## Cholangiopathy

Cholangiocytes represent special epithelial cells that are central to physiologic regulation of biliary function but are also involved in liver regeneration and inflammation. Cholangiopathies result from immune-mediated, genetic or acquired insults and progressive biliary disease leads to end-stage liver disease, including advanced fibrosis and biliary or HCC. Spatial-omics technologies have greatly advanced the study of biliary diseases.^151 152^

Primary sclerosing cholangitis (PSC) is an immune-mediated cholestatic liver disease characterised by bile retention, progressive destruction of biliary tree and fibrosis. In a recent multimodal study, Andrews *et al* constructed a spatially resolved transcriptomic map of PSC using Visium, with spot deconvolution guided by integrated sc-RNA sequencing data.^153^ They observed that during cholangitis development, hepatocytes within fibrotic regions expressing cholangiocyte-associated markers suggested redifferentiation. Immune cell infiltration was observed in PSC scar lesions, and PSC-associated macrophages demonstrated an immune regulatory-like phenotype. In immune-mediated biliary atresia (BA), Xiao *et al* applied Stereo-seq, generating a comprehensive spatial cell atlas, defining 45 distinct subtypes that were organised into 22 discrete tissue niches. Cell communication analysis revealed a pathogenic cascade in which TREM2^+^ MoMFs secrete TNFSF12, engage TNFRSF12A on cholangiocytes, trigger CCL2 production, and thereby recruit additional CCR2^+^ monocytes. Disrupting this TNFSF12–TNFRSF12A–CCL2/CCR2 axis dampens inflammatory infiltration and could represent a therapeutic target for BA.^154^

In biliary injury, Liu *et al* showed that reactive cholangiocytes secrete high levels of ORM2, which localises with neighbouring IBA1+macrophages. ORM2 engages ITPR2 on macrophages, reprogramming them toward a proinflammatory phenotype, which in turn activates HSCs and amplifies fibrogenesis.^155^ Wu *et al* integrated scRNA-seq and stereo-seq, building a spatial and temporal liver atlas with nine zonation layers revealing the injured cholangiocytes as a signalling hub that recruits LAM, drives liver progenitor-like cells into cholangiocytes and modulates hepatocyte regeneration.^156^ Together, these studies emphasise the central role of reactive cholangiocytes in mediating the pathogenesis and progression.

## Perspective

Spatial profiling provides insights into a novel dimension of disease mechanisms that can on the one hand enhance drug development, evaluation of drug efficacy. On the other hand, spatial methods have strong potential for the development of spatial biomarkers and spatial features-based stratification of liver disease that may rapidly enhance diagnosis, stratification and treatment of liver disease.

## Drug development: spatial dimension

Significant heterogeneity of liver disease due to spatial features enables the discovery of spatially defined treatment targets. For example, in HCC patients with tumours surrounded by fibrotic capsules, poor survival may be due to impaired immune cell infiltration and limited drug penetration.^96 157^ Stratifying patients based on their pattern of immune infiltration allows an improved design of therapeutic trials for drug combinations targeting stromal components and enhancing immune infiltration drug delivery.^158^

Novel preclinical models including spheroid or organoid cultures and 3D bioprinting are promising approaches for studying the microenvironment at spatial level, providing a valuable platform for personalised drug screening.^159^ In a proof-of-principle study, Meier *et al* generated organoids from a patient resection with HCC neuroendocrine differentiation and used them for drug response testing. However, organoid screening approaches did not directly translate into clinical efficacy, highlighting current limitations of organoid drug screening.^160^ One possible reason for this discrepancy is that while first generation organoids have a good representation of tumour cells and even tumour heterogeneity, they lack other components of the microenvironment including stroma and low representation of immune cells, which are likely to significantly influence drug efficacy in vivo. Liu *et al* established a cocultures models of HCC derived organoids with cancer-associated fibroblasts (CAFs) and revealed the role of CAFs in mediating treatment resistance.^161^ Further development of organoid systems to recapitulate the spatial organisation of the TME holds promise for improving spatial drug screening and enabling more personalised treatment strategies.

## Time-lapse imaging

The spatial technologies reviewed above are typically used for the profiling at defined timepoints. Methods that allow longitudinal and dynamic mapping would allow another dimension of analysis and understanding. IVM is a method that has shown promise in animal models where it can be used for real-time, in vivo imaging. Recently, Peiseler *et al* used IVM in a model of hepatic fibrosis to identify that bone marrow-derived monocytes can compensate for the loss of KC antibacterial function due to hepatic remodelling by forming KC-like syncytia.^113^ Further examples of insights from this approach in liver research are summarised in a recent review.^162^ However, the translation of IVM approaches into human research is challenging due to the need for the introduction of fluorescent markers. Methods that allow spatial fine-mapping using non-invasive high-resolution imaging technologies (eg, CT, MRI) are evolving towards higher resolution and may become future options for a precise mapping of multiomic spatial data in human settings.

## Identification of spatial biomarkers

Spatial multiomic studies have enabled the identification of spatial immune architectures that correlate with patient prognosis and treatment response, such as TLS, CD8^+^ T cell distribution and tumour capsules, as discussed above. However, identifying spatial biomarkers can be challenging due to issues like incomplete sample capture or the time required for the development of spatial biomarkers. In addition to the observed spatial biomarkers, another promising approach explores the spatial microenvironment changes by inferring from both spatial and temporal dimensions. This approach offers insight into the ongoing evolution of tumours, overcoming the limitation of tissue sections being a fixed representation of a single moment. Kueckelhaus *et al* introduced Spatial Gradient Screening to capture spatial expression dynamics.^46^ By incorporating spatiotemporal dynamics, it appears possible to identify more meaningful and less complex spatial biomarkers at earlier stages.

Vasomics is emerging as another promising field for identifying new spatial biomarkers, as advanced imaging technologies reveal the role pathological vascular features play in disease.^163^ In HCC, the presence of sinusoid-like microvessels is associated with a higher risk of recurrence and poor prognosis, as this microvascular invasion affects tumour perfusion. Further studies are needed to explore the relationship between vascular features and the efficacy of anti-angiogenic therapies.

## Personalised spatial information-based treatment

Spatial multiomic approaches with cellular resolution allow the determination of therapy targets within the microenvironment. In liver cancers, immune classifications based on spatial tumour-immune architecture show promise for patient stratification.^84^ This information may help to tailor therapy regimen to patients based on spatial features. A more detailed understanding of the immune interactions and mechanisms controlling the different immune classes will help develop combination therapies to render patients with a depleted or compartmentalised immune class more responsive to checkpoint therapies. Moreover, while the determination of tumour genetic alterations by NGS sequencing has a role in current personalised decision-making in iCCA (less so in HCC), further assessment of cell-type-specific spatial biomarkers informing about pathways that can be pharmaceutically targeted is expected to provide further detail for identifying suitable personalised treatment options. In addition to oncology, spatial-based features emerging as potential biomarkers for therapies are evolving in inflammatory, infectious and metabolic liver diseases.

## Conclusions

The progress in spatial single-cell profiling technologies represents groundbreaking advances for the study of liver diseases, enabling researchers to understand cellular interactions in distinct liver microenvironments. So far, only a small number of studies have leveraged the potential of current spatial single-cell analysis approaches; however, these seminal papers have reported major insights into key disease mechanisms that depend on spatial biology. We are starting to appreciate that the heterogeneity of liver diseases frequently depends on differences in spatial cellular interactions. These findings have high translational potential and may be harnessed for better patient stratification, diagnostics and drug development, and are likely to impact patient care in the near future. However, further progress in analytical tools to deconvolute spatial features as well as in vivo and in vitro systems to exploit that information for controlled study and perturbation of spatial biology is critically warranted. In sum, novel spatial omics methods now enable a better understanding of the liver in health and disease at a spatially resolved cellular resolution that is critical to address key challenges in hepatology. Results from first studies leveraging these technologies highlight a plethora of novel insights and suggest a transformative role for spatial single-cell technologies in the future of hepatology.

## Supplementary material

10.1136/gutjnl-2024-332105 online supplemental file 1

10.1136/gutjnl-2024-332105 online supplemental file 2

## References

[1] Trefts E, Gannon M, Wasserman DH (2017). The liver. Curr Biol.

[2] Guilliams M, Bonnardel J, Haest B (2022). Spatial proteogenomics reveals distinct and evolutionarily conserved hepatic macrophage niches. Cell.

[3] Calderaro J, Ziol M, Paradis V (2019). Molecular and histological correlations in liver cancer. J Hepatol.

[4] Goodman ZD (2007). Grading and staging systems for inflammation and fibrosis in chronic liver diseases. J Hepatol.

[5] Halpern KB, Shenhav R, Matcovitch-Natan O (2017). Single-cell spatial reconstruction reveals global division of labour in the mammalian liver. Nature.

[6] (2021). Method of the Year 2020: spatially resolved transcriptomics. Nat Methods.

[7] (2024). Method of the Year 2024: spatial proteomics. Nat Methods.

[8] Lewis SM, Asselin-Labat M-L, Nguyen Q (2021). Spatial omics and multiplexed imaging to explore cancer biology. Nat Methods.

[9] Elhanani O, Ben-Uri R, Keren L (2023). Spatial profiling technologies illuminate the tumor microenvironment. Cancer Cell.

[10] Walsh LA, Quail DF (2023). Decoding the tumor microenvironment with spatial technologies. Nat Immunol.

[11] Bressan D, Battistoni G, Hannon GJ (2023). The dawn of spatial omics. Science.

[12] de Souza N, Zhao S, Bodenmiller B (2024). Multiplex protein imaging in tumour biology. Nat Rev Cancer.

[13] Vandereyken K, Sifrim A, Thienpont B (2023). Methods and applications for single-cell and spatial multi-omics. Nat Rev Genet.

[14] Raj A, van den Bogaard P, Rifkin SA (2008). Imaging individual mRNA molecules using multiple singly labeled probes. Nat Methods.

[15] Codeluppi S, Borm LE, Zeisel A (2018). Spatial organization of the somatosensory cortex revealed by osmFISH. Nat Methods.

[16] Xia C, Fan J, Emanuel G (2019). Spatial transcriptome profiling by MERFISH reveals subcellular RNA compartmentalization and cell cycle-dependent gene expression. Proc Natl Acad Sci U S A.

[17] Eng C-HL, Lawson M, Zhu Q (2019). Transcriptome-scale super-resolved imaging in tissues by RNA seqFISH. Nature.

[18] He S, Bhatt R, Brown C (2022). High-plex imaging of RNA and proteins at subcellular resolution in fixed tissue by spatial molecular imaging. Nat Biotechnol.

[19] Janesick A, Shelansky R, Gottscho AD (2023). High resolution mapping of the tumor microenvironment using integrated single-cell, spatial and in situ analysis. Nat Commun.

[20] Wang X, Allen WE, Wright MA (2018). Three-dimensional intact-tissue sequencing of single-cell transcriptional states. Science.

[21] Lee JH, Daugharthy ER, Scheiman J (2015). Fluorescent in situ sequencing (FISSEQ) of RNA for gene expression profiling in intact cells and tissues. Nat Protoc.

[22] Ståhl PL, Salmén F, Vickovic S (2016). Visualization and analysis of gene expression in tissue sections by spatial transcriptomics. Science.

[23] Cho C-S, Xi J, Si Y (2021). Microscopic examination of spatial transcriptome using Seq-Scope. Cell.

[24] Chen A, Liao S, Cheng M (2022). Spatiotemporal transcriptomic atlas of mouse organogenesis using DNA nanoball-patterned arrays. Cell.

[25] Deng Y, Bartosovic M, Ma S (2022). Spatial profiling of chromatin accessibility in mouse and human tissues. Nature.

[26] Deng Y, Bartosovic M, Kukanja P (2022). Spatial-CUT&Tag: Spatially resolved chromatin modification profiling at the cellular level. Science.

[27] Chughtai K, Heeren RMA (2010). Mass spectrometric imaging for biomedical tissue analysis. Chem Rev.

[28] Anderton CR, Gamble LJ (2016). Secondary Ion Mass Spectrometry Imaging of Tissues, Cells, and Microbial Systems. Micros Today.

[29] Gemperline E, Rawson S, Li L (2014). Optimization and Comparison of Multiple MALDI Matrix Application Methods for Small Molecule Mass Spectrometric Imaging. Anal Chem.

[30] Venter A, Sojka PE, Cooks RG (2006). Droplet Dynamics and Ionization Mechanisms in Desorption Electrospray Ionization Mass Spectrometry. Anal Chem.

[31] Nunes JB, Ijsselsteijn ME, Abdelaal T (2024). Integration of mass cytometry and mass spectrometry imaging for spatially resolved single-cell metabolic profiling. Nat Methods.

[32] Hoch T, Schulz D, Eling N (2022). Multiplexed imaging mass cytometry of the chemokine milieus in melanoma characterizes features of the response to immunotherapy. Sci Immunol.

[33] Schulz D, Zanotelli VRT, Fischer JR (2018). Simultaneous Multiplexed Imaging of mRNA and Proteins with Subcellular Resolution in Breast Cancer Tissue Samples by Mass Cytometry. Cell Syst.

[34] Jiang S, Chan CN, Rovira-Clavé X (2022). Combined protein and nucleic acid imaging reveals virus-dependent B cell and macrophage immunosuppression of tissue microenvironments. Immunity.

[35] Gerdes MJ, Sevinsky CJ, Sood A (2013). Highly multiplexed single-cell analysis of formalin-fixed, paraffin-embedded cancer tissue. Proc Natl Acad Sci U S A.

[36] Radtke AJ, Kandov E, Lowekamp B (2020). IBEX: A versatile multiplex optical imaging approach for deep phenotyping and spatial analysis of cells in complex tissues. Proc Natl Acad Sci U S A.

[37] Schubert W, Bonnekoh B, Pommer AJ (2006). Analyzing proteome topology and function by automated multidimensional fluorescence microscopy. Nat Biotechnol.

[38] Lin J-R, Izar B, Wang S (2018). Highly multiplexed immunofluorescence imaging of human tissues and tumors using t-CyCIF and conventional optical microscopes. Elife.

[39] Kinkhabwala A, Herbel C, Pankratz J (2022). MACSima imaging cyclic staining (MICS) technology reveals combinatorial target pairs for CAR T cell treatment of solid tumors. Sci Rep.

[40] Wang Y, Woehrstein JB, Donoghue N (2017). Rapid Sequential in Situ Multiplexing with DNA Exchange Imaging in Neuronal Cells and Tissues. Nano Lett.

[41] Goltsev Y, Samusik N, Kennedy-Darling J (2018). Deep Profiling of Mouse Splenic Architecture with CODEX Multiplexed Imaging. Cell.

[42] Saka SK, Wang Y, Kishi JY (2019). Immuno-SABER enables highly multiplexed and amplified protein imaging in tissues. Nat Biotechnol.

[43] Stack EC, Wang C, Roman KA (2014). Multiplexed immunohistochemistry, imaging, and quantitation: a review, with an assessment of Tyramide signal amplification, multispectral imaging and multiplex analysis. Methods.

[44] Stuart T, Butler A, Hoffman P (2019). Comprehensive Integration of Single-Cell Data. Cell.

[45] Dries R, Zhu Q, Dong R (2021). Giotto: a toolbox for integrative analysis and visualization of spatial expression data. Genome Biol.

[46] Kueckelhaus J, Frerich S, Kada-Benotmane J (2024). Inferring histology-associated gene expression gradients in spatial transcriptomic studies. Nat Commun.

[47] Larsson L, Franzén L, Ståhl PL (2023). Semla: a versatile toolkit for spatially resolved transcriptomics analysis and visualization. Bioinformatics.

[48] Wolf FA, Angerer P, Theis FJ (2018). SCANPY: large-scale single-cell gene expression data analysis. Genome Biol.

[49] Palla G, Spitzer H, Klein M (2022). Squidpy: a scalable framework for spatial omics analysis. Nat Methods.

[50] Pollaris L, Vanneste B, Rombaut B (2024). SPArrOW: a flexible, interactive and scalable pipeline for spatial transcriptomics analysis. Bioinformatics.

[51] Pham D, Tan X, Balderson B (2023). Robust mapping of spatiotemporal trajectories and cell-cell interactions in healthy and diseased tissues. Nat Commun.

[52] Windhager J, Zanotelli VRT, Schulz D (2023). An end-to-end workflow for multiplexed image processing and analysis. Nat Protoc.

[53] Palmer A, Phapale P, Chernyavsky I (2017). FDR-controlled metabolite annotation for high-resolution imaging mass spectrometry. Nat Methods.

[54] Yue L, Liu F, Hu J (2023). A guidebook of spatial transcriptomic technologies, data resources and analysis approaches. Comput Struct Biotechnol J.

[55] Valihrach L, Zucha D, Abaffy P (2024). A practical guide to spatial transcriptomics. Mol Aspects Med.

[56] Milosevic V (2023). Different approaches to Imaging Mass Cytometry data analysis. Bioinformatics Advances.

[57] Berg S, Kutra D, Kroeger T (2019). ilastik: interactive machine learning for (bio)image analysis. Nat Methods.

[58] Stirling DR, Swain-Bowden MJ, Lucas AM (2021). CellProfiler 4: improvements in speed, utility and usability. BMC Bioinformatics.

[59] Rueden CT, Schindelin J, Hiner MC (2017). ImageJ2: ImageJ for the next generation of scientific image data. BMC Bioinformatics.

[60] Stringer C, Wang T, Michaelos M (2021). Cellpose: a generalist algorithm for cellular segmentation. Nat Methods.

[61] Bannon D, Moen E, Schwartz M (2021). DeepCell Kiosk: scaling deep learning–enabled cellular image analysis with Kubernetes. Nat Methods.

[62] Kleshchevnikov V, Shmatko A, Dann E (2022). Cell2location maps fine-grained cell types in spatial transcriptomics. Nat Biotechnol.

[63] Biancalani T, Scalia G, Buffoni L (2021). Deep learning and alignment of spatially resolved single-cell transcriptomes with Tangram. Nat Methods.

[64] Cable DM, Murray E, Zou LS (2022). Robust decomposition of cell type mixtures in spatial transcriptomics. Nat Biotechnol.

[65] Chidester B, Zhou T, Alam S (2023). SPICEMIX enables integrative single-cell spatial modeling of cell identity. Nat Genet.

[66] Miller BF, Huang F, Atta L (2022). Reference-free cell type deconvolution of multi-cellular pixel-resolution spatially resolved transcriptomics data. Nat Commun.

[67] Vicari M, Mirzazadeh R, Nilsson A (2024). Spatial multimodal analysis of transcriptomes and metabolomes in tissues. Nat Biotechnol.

[68] Ravi VM, Will P, Kueckelhaus J (2022). Spatially resolved multi-omics deciphers bidirectional tumor-host interdependence in glioblastoma. Cancer Cell.

[69] Sung H, Ferlay J, Siegel RL (2021). Global Cancer Statistics 2020: GLOBOCAN Estimates of Incidence and Mortality Worldwide for 36 Cancers in 185 Countries. CA Cancer J Clin.

[70] Finn RS, Qin S, Ikeda M (2020). Atezolizumab plus Bevacizumab in Unresectable Hepatocellular Carcinoma. N Engl J Med.

[71] Cheng A-L, Qin S, Ikeda M (2022). Updated efficacy and safety data from IMbrave150: Atezolizumab plus bevacizumab vs. sorafenib for unresectable hepatocellular carcinoma. J Hepatol.

[72] Ren Z, Xu J, Bai Y (2021). Sintilimab plus a bevacizumab biosimilar (IBI305) versus sorafenib in unresectable hepatocellular carcinoma (ORIENT-32): a randomised, open-label, phase 2-3 study. Lancet Oncol.

[73] Abou-Alfa GK, Lau G, Kudo M (2022). Tremelimumab plus Durvalumab in Unresectable Hepatocellular Carcinoma. *NEJM Evid*.

[74] Yau T, Kang Y-K, Kim T-Y (2020). Efficacy and Safety of Nivolumab Plus Ipilimumab in Patients With Advanced Hepatocellular Carcinoma Previously Treated With Sorafenib: The CheckMate 040 Randomized Clinical Trial. JAMA Oncol.

[75] Oh D-Y, Ruth He A, Qin S (2022). Durvalumab plus Gemcitabine and Cisplatin in Advanced Biliary Tract Cancer. *NEJM Evid*.

[76] Xue R, Zhang Q, Cao Q (2022). Liver tumour immune microenvironment subtypes and neutrophil heterogeneity. Nature New Biol.

[77] Ma L, Wang L, Khatib SA (2021). Single-cell atlas of tumor cell evolution in response to therapy in hepatocellular carcinoma and intrahepatic cholangiocarcinoma. J Hepatol.

[78] Zhang Q, He Y, Luo N (2019). Landscape and Dynamics of Single Immune Cells in Hepatocellular Carcinoma. Cell.

[79] Ma L, Hernandez MO, Zhao Y (2019). Tumor Cell Biodiversity Drives Microenvironmental Reprogramming in Liver Cancer. Cancer Cell.

[80] Bian J, Lin J, Long J (2020). T lymphocytes in hepatocellular carcinoma immune microenvironment: insights into human immunology and immunotherapy. Am J Cancer Res.

[81] Greten TF, Villanueva A, Korangy F (2023). Biomarkers for immunotherapy of hepatocellular carcinoma. Nat Rev Clin Oncol.

[82] Ho DW-H, Tsui Y-M, Chan L-K (2021). Single-cell RNA sequencing shows the immunosuppressive landscape and tumor heterogeneity of HBV-associated hepatocellular carcinoma. Nat Commun.

[83] Barsch M, Salié H, Schlaak AE (2022). T-cell exhaustion and residency dynamics inform clinical outcomes in hepatocellular carcinoma. J Hepatol.

[84] Salié H, Wischer L, D’Alessio A (2025). Spatial single-cell profiling and neighbourhood analysis reveal the determinants of immune architecture connected to checkpoint inhibitor therapy outcome in hepatocellular carcinoma. Gut.

[85] Myojin Y, Babaei S, Trehan R (2025). Multiomics analysis of immune correlatives in hepatocellular carcinoma patients treated with tremelimumab plus durvalumab. Gut.

[86] Jia G, He P, Dai T (2025). Spatial immune scoring system predicts hepatocellular carcinoma recurrence. Nature.

[87] Magen A, Hamon P, Fiaschi N (2023). Intratumoral dendritic cell–CD4+ T helper cell niches enable CD8+ T cell differentiation following PD-1 blockade in hepatocellular carcinoma. Nat Med.

[88] Ho WJ, Zhu Q, Durham J (2021). Neoadjuvant Cabozantinib and Nivolumab Converts Locally Advanced HCC into Resectable Disease with Enhanced Antitumor Immunity. *Nat Cancer*.

[89] Zhang S, Yuan L, Danilova L (2023). Spatial transcriptomics analysis of neoadjuvant cabozantinib and nivolumab in advanced hepatocellular carcinoma identifies independent mechanisms of resistance and recurrence. Genome Med.

[90] Meylan M, Petitprez F, Becht E (2022). Tertiary lymphoid structures generate and propagate anti-tumor antibody-producing plasma cells in renal cell cancer. Immunity.

[91] Andersson A, Larsson L, Stenbeck L (2021). Spatial deconvolution of HER2-positive breast cancer delineates tumor-associated cell type interactions. Nat Commun.

[92] Shu DH, Ho WJ, Kagohara LT (2024). Immunotherapy response induces divergent tertiary lymphoid structure morphologies in hepatocellular carcinoma. Nat Immunol.

[93] Wu L, Yan J, Bai Y (2023). An invasive zone in human liver cancer identified by Stereo-seq promotes hepatocyte-tumor cell crosstalk, local immunosuppression and tumor progression. Cell Res.

[94] Ruf B, Bruhns M, Babaei S (2023). Tumor-associated macrophages trigger MAIT cell dysfunction at the HCC invasive margin. Cell.

[95] Lim JH, Choi D, Park CK (2006). Encapsulated hepatocellular carcinoma: CT-pathologic correlations. Eur Radiol.

[96] Liu Y, Xun Z, Ma K (2023). Identification of a tumour immune barrier in the HCC microenvironment that determines the efficacy of immunotherapy. J Hepatol.

[97] Henderson NC, Rieder F, Wynn TA (2020). Fibrosis: from mechanisms to medicines. Nature.

[98] Schwabe RF, Brenner DA (2025). Hepatic stellate cells: balancing homeostasis, hepatoprotection and fibrogenesis in health and disease. Nat Rev Gastroenterol Hepatol.

[99] Sugimoto A, Saito Y, Wang G (2025). Hepatic stellate cells control liver zonation, size and functions via R-spondin 3. Nature.

[100] Filliol A, Saito Y, Nair A (2022). Opposing roles of hepatic stellate cell subpopulations in hepatocarcinogenesis. Nature.

[101] Mederacke I, Hsu CC, Troeger JS (2013). Fate tracing reveals hepatic stellate cells as dominant contributors to liver fibrosis independent of its aetiology. Nat Commun.

[102] Tsuchida T, Friedman SL (2017). Mechanisms of hepatic stellate cell activation. Nat Rev Gastroenterol Hepatol.

[103] Wang S, Li K, Pickholz E (2023). An autocrine signaling circuit in hepatic stellate cells underlies advanced fibrosis in nonalcoholic steatohepatitis. Sci Transl Med.

[104] Intercept announces outcome of FDA advisory committee meeting for obeticholic acid as a treatment for pre-cirrhotic fibrosis due to NASH. https://www.natap.org/2023/HCV/060123_01.htm.

[105] Tacke F, Puengel T, Loomba R (2023). An integrated view of anti-inflammatory and antifibrotic targets for the treatment of NASH. J Hepatol.

[106] Chung BK, Øgaard J, Reims HM (2022). Spatial transcriptomics identifies enriched gene expression and cell types in human liver fibrosis. *Hepatol Commun*.

[107] Li J-Z, Yang L, Xiao M-X (2025). Spatial and Single-Cell Transcriptomics Reveals the Regional Division of the Spatial Structure of MASH Fibrosis. Liver Int.

[108] Khanal S, Liu Y, Bamidele AO (2024). Glycolysis in hepatic stellate cells coordinates fibrogenic extracellular vesicle release spatially to amplify liver fibrosis. Sci Adv.

[109] Scott CL, Zheng F, De Baetselier P (2016). Bone marrow-derived monocytes give rise to self-renewing and fully differentiated Kupffer cells. Nat Commun.

[110] Krenkel O, Puengel T, Govaere O (2018). Therapeutic inhibition of inflammatory monocyte recruitment reduces steatohepatitis and liver fibrosis. Hepatology.

[111] Krenkel O, Hundertmark J, Abdallah AT (2020). Myeloid cells in liver and bone marrow acquire a functionally distinct inflammatory phenotype during obesity-related steatohepatitis. Gut.

[112] Pellicoro A, Ramachandran P, Iredale JP (2014). Liver fibrosis and repair: immune regulation of wound healing in a solid organ. Nat Rev Immunol.

[113] Peiseler M, Araujo David B, Zindel J (2023). Kupffer cell-like syncytia replenish resident macrophage function in the fibrotic liver. Science.

[114] Tran S, Baba I, Poupel L (2020). Impaired Kupffer Cell Self-Renewal Alters the Liver Response to Lipid Overload during Non-alcoholic Steatohepatitis. Immunity.

[115] Seidman JS, Troutman TD, Sakai M (2020). Niche-Specific Reprogramming of Epigenetic Landscapes Drives Myeloid Cell Diversity in Nonalcoholic Steatohepatitis. Immunity.

[116] Remmerie A, Martens L, Thoné T (2020). Osteopontin Expression Identifies a Subset of Recruited Macrophages Distinct from Kupffer Cells in the Fatty Liver. Immunity.

[117] Ramachandran P, Dobie R, Wilson-Kanamori JR (2019). Resolving the fibrotic niche of human liver cirrhosis at single-cell level. Nature.

[118] Hendrikx T, Porsch F, Kiss MG (2022). Soluble TREM2 levels reflect the recruitment and expansion of TREM2^+^ macrophages that localize to fibrotic areas and limit NASH. J Hepatol.

[119] Guillot A, Winkler M, Silva Afonso M (2023). Mapping the hepatic immune landscape identifies monocytic macrophages as key drivers of steatohepatitis and cholangiopathy progression. Hepatology.

[120] Thomson AW, Knolle PA (2010). Antigen-presenting cell function in the tolerogenic liver environment. Nat Rev Immunol.

[121] Iannacone M, Guidotti LG (2022). Immunobiology and pathogenesis of hepatitis B virus infection. Nat Rev Immunol.

[122] Traum D, Wang YJ, Schwarz KB (2021). Highly multiplexed 2-dimensional imaging mass cytometry analysis of HBV-infected liver. JCI Insight.

[123] Aggarwal A, Odorizzi PM, Brodbeck J (2023). Intrahepatic quantification of HBV antigens in chronic hepatitis B reveals heterogeneity and treatment-mediated reductions in HBV core-positive cells. *JHEP Reports*.

[124] van Buuren N, Ramirez R, Turner S (2022). Characterization of the liver immune microenvironment in liver biopsies from patients with chronic HBV infection. *JHEP Rep*.

[125] Yu X, Gong Q, Yu D (2024). Spatial transcriptomics reveals a low extent of transcriptionally active hepatitis B virus integration in patients with HBsAg loss. Gut.

[126] Pita-Juarez Y, Karagkouni D, Kalavros N (2022). A single-nucleus and spatial transcriptomic atlas of the COVID-19 liver reveals topological, functional, and regenerative organ disruption in patients. bioRxiv.

[127] Delorey TM, Ziegler CGK, Heimberg G (2021). COVID-19 tissue atlases reveal SARS-CoV-2 pathology and cellular targets. Nature.

[128] Palla P, Vergadis C, Sakellariou S (2022). Letter to the editor: Autoimmune hepatitis after COVID-19 vaccination: A rare adverse effect?. *Hepatology*.

[129] Bril F, Al Diffalha S, Dean M (2021). Autoimmune hepatitis developing after coronavirus disease 2019 (COVID-19) vaccine: Causality or casualty?. J Hepatol.

[130] Lodato F, Larocca A, D’Errico A (2021). An unusual case of acute cholestatic hepatitis after m-RNABNT162b2 (Comirnaty) SARS-CoV-2 vaccine: Coincidence, autoimmunity or drug-related liver injury. J Hepatol.

[131] Boettler T, Csernalabics B, Salié H (2022). SARS-CoV-2 vaccination can elicit a CD8 T-cell dominant hepatitis. J Hepatol.

[132] Uzun S, Zinner CP, Beenen AC (2023). Morphologic and molecular analysis of liver injury after SARS-CoV-2 vaccination reveals distinct characteristics. J Hepatol.

[133] Röttele F, Zollner A, Mogler C (2025). Characteristic immune cell interactions in livers of children with acute hepatitis revealed by spatial single-cell analysis identify a possible postacute sequel of COVID-19. Gut.

[134] Kang SWS, Cunningham RP, Miller CB (2024). A spatial map of hepatic mitochondria uncovers functional heterogeneity shaped by nutrient-sensing signaling. Nat Commun.

[135] Xiong X, Kuang H, Ansari S (2019). Landscape of Intercellular Crosstalk in Healthy and NASH Liver Revealed by Single-Cell Secretome Gene Analysis. Mol Cell.

[136] Jaitin DA, Adlung L, Thaiss CA (2019). Lipid-Associated Macrophages Control Metabolic Homeostasis in a Trem2-Dependent Manner. Cell.

[137] Deczkowska A, David E, Ramadori P (2021). XCR1^+^ type 1 conventional dendritic cells drive liver pathology in non-alcoholic steatohepatitis. Nat Med.

[138] Dudek M, Pfister D, Donakonda S (2021). Auto-aggressive CXCR6^+^ CD8 T cells cause liver immune pathology in NASH. Nature.

[139] Ochoa-Rios S, O’Connor IP, Kent LN (2022). Imaging Mass Spectrometry Reveals Alterations in N-Linked Glycosylation That Are Associated With Histopathological Changes in Nonalcoholic Steatohepatitis in Mouse and Human. *Mol Cell Proteomics*.

[140] Seubnooch P, Montani M, Tsouka S (2023). Characterisation of hepatic lipid signature distributed across the liver zonation using mass spectrometry imaging. *JHEP Rep*.

[141] Hall Z, Bond NJ, Ashmore T (2017). Lipid zonation and phospholipid remodeling in nonalcoholic fatty liver disease. Hepatology.

[142] Seubnooch P, Montani M, Dufour JF (2024). Spatial lipidomics reveals zone-specific hepatic lipid alteration and remodeling in metabolic dysfunction-associated steatohepatitis. J Lipid Res.

[143] Zhou Y, Zhao Y, Carbonaro M (2024). Perturbed liver gene zonation in a mouse model of non-alcoholic steatohepatitis. Metab Clin Exp.

[144] Michalopoulos GK, Bhushan B (2021). Liver regeneration: biological and pathological mechanisms and implications. Nat Rev Gastroenterol Hepatol.

[145] Chen F, Schönberger K, Tchorz JS (2023). Distinct hepatocyte identities in liver homeostasis and regeneration. *JHEP Rep*.

[146] Yanger K, Zong Y, Maggs LR (2013). Robust cellular reprogramming occurs spontaneously during liver regeneration. Genes Dev.

[147] Pu W, Zhu H, Zhang M (2023). Bipotent transitional liver progenitor cells contribute to liver regeneration. Nat Genet.

[148] Font-Burgada J, Shalapour S, Ramaswamy S (2015). Hybrid Periportal Hepatocytes Regenerate the Injured Liver without Giving Rise to Cancer. Cell.

[149] Lin Y-H, Wei Y, Zeng Q (2023). IGFBP2 expressing midlobular hepatocytes preferentially contribute to liver homeostasis and regeneration. Cell Stem Cell.

[150] Matchett KP, Wilson-Kanamori JR, Portman JR (2024). Multimodal decoding of human liver regeneration. Nature.

[151] Banales JM, Huebert RC, Karlsen T (2019). Cholangiocyte pathobiology. Nat Rev Gastroenterol Hepatol.

[152] Jalan-Sakrikar N, Guicciardi ME, O’Hara SP (2024). Central role for cholangiocyte pathobiology in cholestatic liver diseases. Hepatology.

[153] Andrews TS, Nakib D, Perciani CT (2024). Single-cell, single-nucleus, and spatial transcriptomics characterization of the immunological landscape in the healthy and PSC human liver. J Hepatol.

[154] Xiao M-H, Ma D, Wu S (2025). Integrative single-cell and spatial transcriptomic analyses identify a pathogenic cholangiocyte niche and TNFRSF12A as therapeutic target for biliary atresia. Hepatology.

[155] Liu H, Yin G, Franco Leonardi B (2025). Reactive cholangiocyte-derived ORM2 drives a pathogenic modulation of the injured biliary niche through macrophage reprogramming. Gut.

[156] Wu B, Shentu X, Nan H (2024). A spatiotemporal atlas of cholestatic injury and repair in mice. Nat Genet.

[157] Wu R, Guo W, Qiu X (2021). Comprehensive analysis of spatial architecture in primary liver cancer. Sci Adv.

[158] Chitty JL, Yam M, Perryman L (2023). A first-in-class pan-lysyl oxidase inhibitor impairs stromal remodeling and enhances gemcitabine response and survival in pancreatic cancer. *Nat Cancer*.

[159] Marsee A, Roos FJM, Verstegen MMA (2021). Building consensus on definition and nomenclature of hepatic, pancreatic, and biliary organoids. Cell Stem Cell.

[160] Meier M-A, Nuciforo S, Coto-Llerena M (2022). Patient-derived tumor organoids for personalized medicine in a patient with rare hepatocellular carcinoma with neuroendocrine differentiation: a case report. Commun Med (Lond).

[161] Liu J, Li P, Wang L (2021). Cancer-Associated Fibroblasts Provide a Stromal Niche for Liver Cancer Organoids That Confers Trophic Effects and Therapy Resistance. Cell Mol Gastroenterol Hepatol.

[162] Wang Y, Heymann F, Peiseler M (2024). Intravital imaging: dynamic insights into liver immunity in health and disease. Gut.

[163] Wang C, Felli E, Fallowfield JA (2025). Vasomics of the liver. Gut.

