# Supplementary material for: Spatial single-cell omics: new insights into liver diseases
Source: Gut. 2025 Aug 5;75(6):e332105. doi: 10.1136/gutjnl-2024-332105 (PMC13217129; doi:10.1136/gutjnl-2024-332105)
Supplement: online supplemental file 2 [file gutjnl-75-6-s002.pptx]

## Slide 1
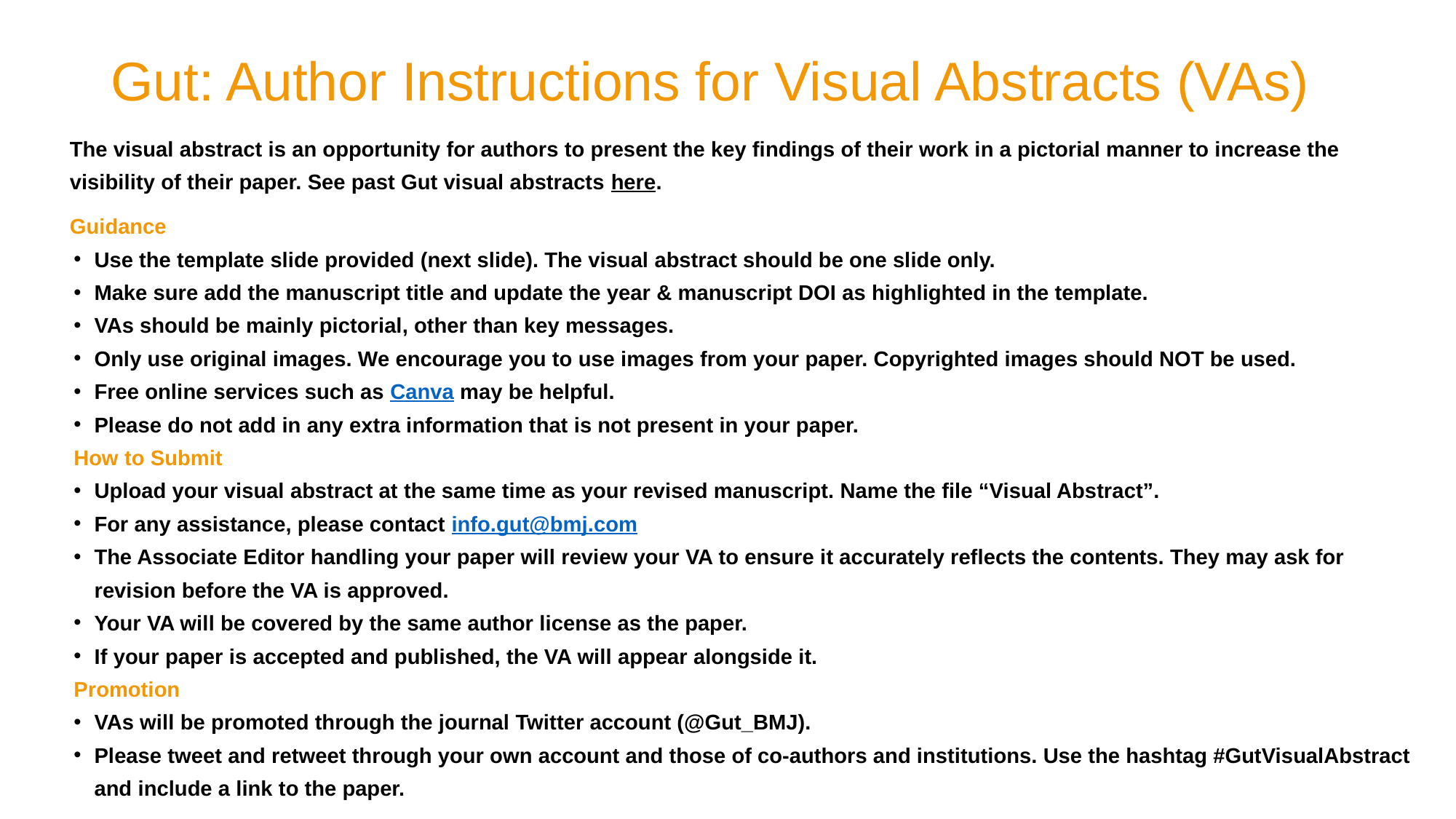

# Gut: Author Instructions for Visual Abstracts (VAs)
The visual abstract is an opportunity for authors to present the key findings of their work in a pictorial manner to increase the visibility of their paper. See past Gut visual abstracts here.
Guidance
Use the template slide provided (next slide). The visual abstract should be one slide only.
Make sure add the manuscript title and update the year & manuscript DOI as highlighted in the template.
VAs should be mainly pictorial, other than key messages.
Only use original images. We encourage you to use images from your paper. Copyrighted images should NOT be used.
Free online services such as Canva may be helpful.
Please do not add in any extra information that is not present in your paper.
How to Submit
Upload your visual abstract at the same time as your revised manuscript. Name the file “Visual Abstract”.
For any assistance, please contact info.gut@bmj.com
The Associate Editor handling your paper will review your VA to ensure it accurately reflects the contents. They may ask for revision before the VA is approved.
Your VA will be covered by the same author license as the paper.
If your paper is accepted and published, the VA will appear alongside it.
Promotion
VAs will be promoted through the journal Twitter account (@Gut_BMJ).
Please tweet and retweet through your own account and those of co-authors and institutions. Use the hashtag #GutVisualAbstract and include a link to the paper.

## Slide 2
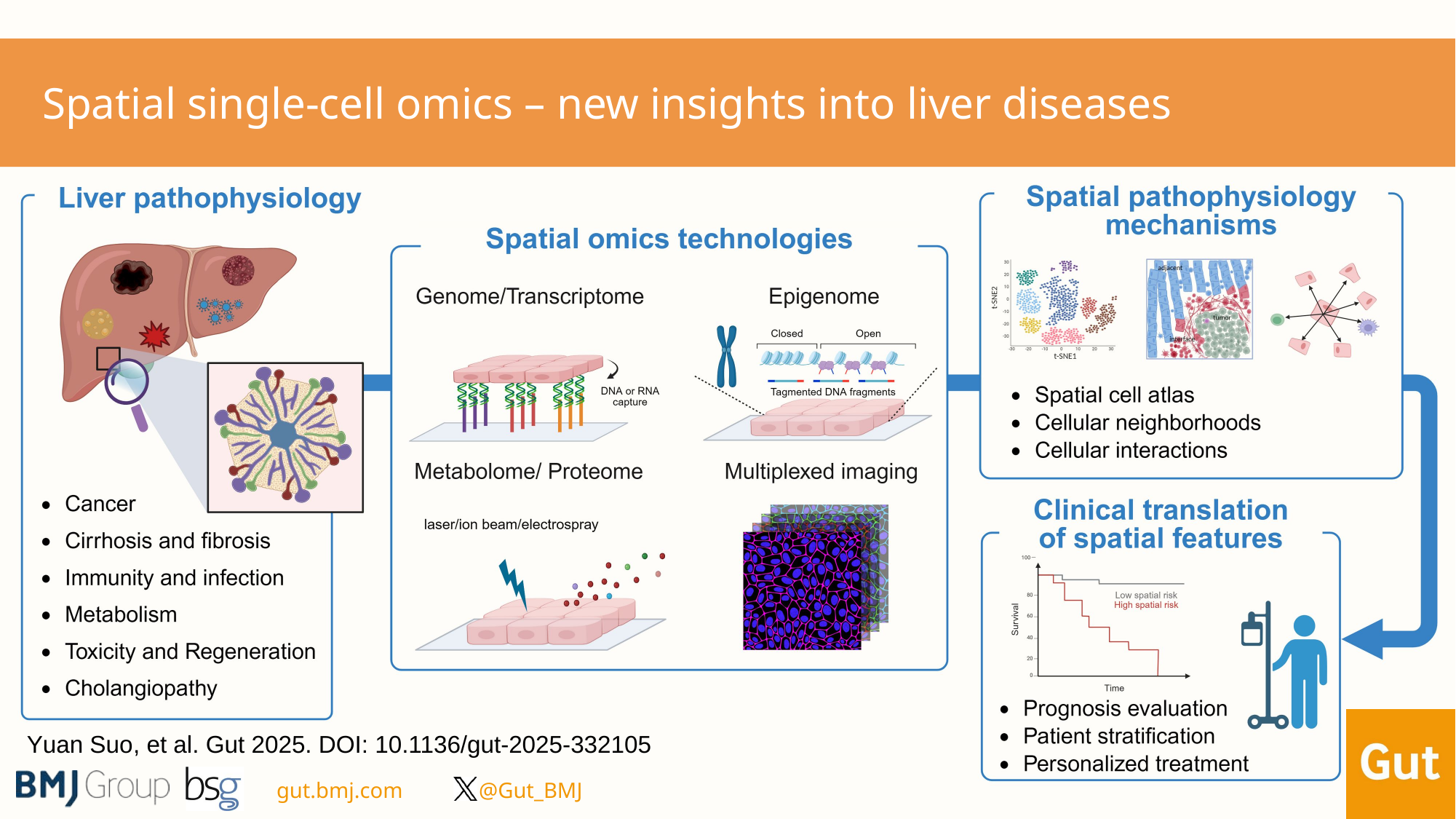

Spatial single-cell omics – new insights into liver diseases
Yuan Suo, et al. Gut 2025. DOI: 10.1136/gut-2025-332105
gut.bmj.com
@Gut_BMJ
